# Global Geo-Pharmacogenomics: Environmental Mutational Signatures Drive Population-Level Heterogeneity in Anticancer Drug Response

**DOI:** 10.3390/jox16030087

**Published:** 2026-05-18

**Authors:** Janiel Jawahar, Samuel James

**Affiliations:** Department of Civil Engineering, Hindustan Institute of Technology and Science, Rajiv Gandhi Salai (OMR), Padur, Chennai 603103, India; jsamuel@hindustanuniv.ac.in

**Keywords:** geo-pharmacogenomics, mutational signatures, environmental exposome, drug response, precision oncology, remote sensing, GIS

## Abstract

The interplay between the environmental exposome and the cancer genome remains a critical gap in precision oncology. While somatic mutational signatures—genomic fossils imprinted by exposures such as ultraviolet radiation; tobacco smoke; and industrial pollutants—are well characterised for their etiological significance; their functional impact on therapeutic efficacy remains largely unexplored. We hypothesised that these environmental genomic scars induce distinct pharmacogenomic vulnerabilities and resistance mechanisms that vary by geographical exposure patterns. This study employs two complementary analytical frameworks. First, a linear regression-based pharmacogenomic screen across four datasets (GDSC1, GDSC2, CTRP, CCLE; 1001 cell lines, 31 cancer types) identified 608 statistically significant (*p* < 0.01) mutational signature–drug interactions, revealing that UV-associated signature SBS7a is associated with broad-spectrum therapeutic resistance, including to BRAF inhibitors (PLX-4720, *p* < 10^−4^), while pollution-driven oxidative stress (SBS18) is associated with sensitivity to p38 MAPK inhibition (VX-702, r = −0.45, *p* < 10^−9^). Second, an XGBoost predictive model trained exclusively on 33,679 GDSC2 records using a 1265-feature matrix integrating 40 SBS signatures, drug chemistry descriptors, proteomic features, and two satellite-derived environmental variables (NASA PM_2.5_ and UV)—achieved R^2^ = 0.7973 on a 20% holdout set (grouped cross-validation R^2^ = 0.7296). SHAP analysis revealed that satellite-derived PM_2.5_ (Zone_PM25) ranked 7th of 1265 features, exceeding all 40 individual SBS mutational signatures. Synthesising these findings with satellite-derived atmospheric data, we constructed an exploratory spatially interpolated risk surface spanning 122 nations, generating the hypothesis that uniform drug efficacy assumptions may not apply globally. These findings suggest that a patient’s environmental exposure history may constitute a measurable pharmacogenomic variable. This exploratory framework warrants validation in independent datasets and with individual-level geographic data before clinical application.

## 1. Introduction

The global burden of cancer remains one of the most formidable challenges to contemporary public health, characterised by a complex, multifactorial aetiology that transcends simple genetic determinism [1]. While somatic mutations are the fundamental drivers of oncogenesis, the vast majority of these genomic alterations arise from a synergistic interplay between endogenous biological processes and exogenous environmental exposures [2,3]. Epidemiological frameworks utilising Population Attributable Risk (PAR) estimates have long aimed to quantify the impact of specific exposures; yet, in the domain of environmental carcinogenesis, the use of PAR has remained contentious and subject to ongoing revision [4,5].

For nearly half a century, the field of cancer epidemiology was heavily influenced by the seminal 1981 report by Doll and Peto [6,7]. In their landmark analysis, “The Causes of Cancer: Quantitative Estimates of Avoidable Risks of Cancer in the United States Today,” they estimated that tobacco use accounted for approximately 30% of cancer deaths, while diet contributed a further 35% [6,7]. In contrast, the PAR for environmental pollution was estimated at merely 2% (range: 1–5%) and occupational exposures at approximately 4% (range: 2–8%) [6]. These conservative figures became deeply embedded in oncological dogma for decades [8]. The Doll and Peto estimates were derived from mortality data predating widespread identification of many modern industrial carcinogens [9,10]. Their definition of “pollution” was relatively narrow, and they explicitly acknowledged the difficulty of quantifying risks from ubiquitous, low-level exposures [11]. Modern epidemiological re-evaluations argue that the “2% estimate” masks significant heterogeneity across cancer types and populations [4,12,13]. When viewed through organ-specific lenses, the environmental dependency becomes strikingly pronounced. A prime example is malignant mesothelioma, where approximately 80% of cases are directly attributable to asbestos exposure [14,15]. Attributable risk for pleural mesothelioma among men with occupational exposure can exceed 88% [16,17]. This underscores the imperative of moving beyond aggregate statistics toward organ-specific and exposure-specific models that more faithfully represent the true etiological weight of the exposome [2,18].

A pivotal recalibration occurred in 2013, when the International Agency for Research on Cancer (IARC) officially classified outdoor air pollution and particulate matter (PM) as Group 1 human carcinogens [19,20]. This classification positioned ambient air pollution alongside tobacco smoke, asbestos, and plutonium [19]. The evaluation was supported by large-scale cohort studies, including the American Cancer Society Cancer Prevention Study-II (CPS-II), which enrolled over 1.2 million participants and linked long-term exposure to fine particulate matter (PM_2.5_) with increased lung cancer mortality [21,22]. The European Study of Cohorts for Air Pollution Effects (ESCAPE) demonstrated a statistically significant association between particulate matter and lung adenocarcinoma even at concentrations below EU limit values [23,24]. Mechanistically, fine particulate matter (PM_2.5_) penetrates deep into pulmonary alveoli, inducing chronic inflammation and sustained oxidative stress via generation of reactive oxygen species (ROS) that directly damage DNA and disrupt intracellular signalling [25]. Polycyclic aromatic hydrocarbons (PAHs) adsorbed onto particle surfaces enter the airways and form bulky DNA adducts that interfere with replication fidelity. These processes drive inactivation of tumour suppressor genes such as TP53 and activation of oncogenes, thereby advancing the somatic evolution of the cancer genome [25,26].

The impact of environmental carcinogens is not uniform across global populations. The interaction between the exposome and the genome is modulated by the host’s germline genetic architecture, producing population-specific susceptibilities [27,28]. For example, the mutational landscape of lung cancer in never-smokers exhibits distinct ethnic variations: EGFR mutations are the predominant driver in East Asian patients, occurring in 50–60% of cases [29], but are less frequent in European and Latin American populations; in the latter, genomic studies reveal distinct mutational signature landscapes [28,30] and lower smoking-associated signature (SBS4) activity [31]. Genomic studies of admixed Latin American populations further reveal that Native American ancestry is associated with specific somatic landscapes, including a lower tumour mutation burden and distinct driver mutation frequencies, independent of smoking history [27,31]. These disparities suggest that population-specific variation in carcinogen metabolism and DNA repair pathways alters how environmental insults are genomically inscribed [27,32]. The field is consequently evolving toward “environmental oncology”, integrating geospatial science with advanced epidemiological methods to enable large-scale exposure assessments and to discern signatures of environmental agents in primary human tumours [33,34,35].

The conceptualisation of mutational signatures represents a paradigm shift in oncology, transitioning focus from individual driver mutations to the holistic “archaeological record” inscribed within a cancer genome [36,37]. Every somatic mutation is the product of a specific mutational process arising from exogenous carcinogen exposure, endogenous metabolic instability, or DNA repair deficiency, leaving a distinct genomic imprint defined by base substitution types and their immediate trinucleotide sequence context [38]. Early somatic mutation studies were confined to frequently mutated single genes such as TP53 or KRAS [39]. Next-generation sequencing (NGS) enabled genome-wide analyses, providing the statistical power to resolve complex mutational patterns [40]. The foundational breakthrough was a mathematical framework treating a cancer genome as a weighted sum of discrete mutational signatures, pioneered by Alexandrov, Stratton, and colleagues using non-negative matrix factorisation (NMF) [41,42]. The Catalogue of Somatic Mutations in Cancer (COSMIC) has become the definitive repository for these signatures [43]. Early iterations identified approximately 30 distinct signatures across 40 cancer types [36]; SBS1, for example, is attributed to the spontaneous deamination of 5-methylcytosine at CpG dinucleotides, a clock-like process that accumulates linearly with the age at tumour diagnosis [44].

Among environmentally linked signatures, SBS4, characterised by C>A transversions, is linked to tobacco smoking and found predominantly in lung and head-and-neck cancers [45]. SBS7a and SBS7b, characterised by C>T transitions at dipyrimidine sites, are the genomic fingerprints of ultraviolet (UV) light exposure, found predominantly in melanoma [46]. SBS2 and SBS13 reflect the aberrant activity of Apolipoprotein B mRNA Editing Enzyme Catalytic subunit (APOBEC) cytidine deaminases [47,48]. As sequencing expanded to whole-genome sequencing (WGS), notably through the Pan-Cancer Analysis of Whole Genomes (PCAWG) consortium, signature resolution improved substantially [49,50]. The latest COSMIC catalogue (v3 and beyond) encompasses over 60 SBS signatures alongside doublet-base substitutions (DBS) and small insertions/deletions (ID) [51]. A landmark experimental compendium by Kucab et al. (2019) exposed human-induced pluripotent stem cells (iPSCs) to 79 known or suspected carcinogens, establishing direct causal links between specific environmental agents and signatures observed in human tumours [52]. Despite this progress, numerous signatures remain of cryptic or unknown origin, highlighting unidentified mutational processes actively sculpting the cancer genome [53]. Robustness of signature extraction is further complicated by subclonal heterogeneity and variability introduced by different sequencing technologies and bioinformatic pipelines [54,55].

Historically, cancer genomics has been predominantly “driver-centric,” focusing on mutations in TP53, KRAS, BRAF, and EGFR that confer a selective growth advantage [56,57]. This focus has relegated the vast majority of somatic alterations termed “passenger mutations” to the status of genomic noise [56,57,58]. Recent evidence challenges this view: the “mini-driver” model proposes that while individual passengers may not drive oncogenesis independently, their cumulative effect can modulate tumour progression under selective pressures of chemotherapy or metastasis [59,60]. More importantly, because passenger mutations are not subject to positive selection, they provide a statistically robust and unbiased record of the mutational processes active throughout the cell’s lineage [36,61]. Pan-cancer analyses from the PCAWG consortium (over 2500 genomes) have demonstrated that the aggregate impact of putative passenger mutations provides significant predictive power for distinguishing cancer from non-cancer phenotypes and correlates with patient survival times [62,63,64]. In the context of environmental epidemiology, driver mutations reveal what the cancer is currently doing, while passenger mutations reveal how it arrived there via UV exposure, smoking, or oxidative stress, enabling a more comprehensive determination of individual mutagen contributions [61].

The frontier of precision oncology lies in the prospective application of mutational signatures to predict therapeutic vulnerability [65]. Traditionally, pharmacogenomics has focused on single-gene markers (EGFR, BRAF V600E), yet the heterogeneity of clinical responses indicates these are insufficient to fully explain drug sensitivity and resistance [65,66]. Large-scale initiatives, including the Genomics of Drug Sensitivity in Cancer (GDSC) and the Cancer Cell Line Encyclopedia (CCLE), have begun to systematically correlate genomic alterations with drug response across hundreds of cancer cell lines [66,67,68]. Drug sensitivity, measured as IC50 values, is influenced by point mutations, copy number variations, transcriptomic profiles, and epigenetic states [69]. Mutational signatures themselves are increasingly recognised as direct indicators of drug mechanism and efficacy. The Kucab et al. experimental compendium demonstrated that chemotherapeutic drugs, including cisplatin and carboplatin, leave distinct mutational footprints, evidence of DNA repair machinery engagement or failure [52,70]. Clinically, the HRDetect framework utilises specific mutational signatures (including SBS3) to predict BRCA1/2 deficiency, identifying patients who may benefit from PARP inhibitors even without traditional germline testing [71]. This establishes the critical precedent that the shape of mutation burden, not merely its location, dictates therapeutic response.

Specific case studies illustrate the functional impact of environmental genomic scars on therapeutic outcomes. The clinical application of BRAF(V600E) inhibitors such as vemurafenib (PLX4032) exposes both the promise and limitations of single gene biomarker strategies [72]. Approximately 50% of primary melanomas harbour the BRAF(V600E) mutation [73], a lesion with a strong etiological link to the UV-induced signature SBS7a, yet therapeutic response to BRAF inhibition is strikingly heterogeneous across tissue lineages [72,74]. The “melanoma-CRC paradox” illustrates this: BRAF(V600E)-mutant melanoma shows high sensitivity to vemurafenib, while colorectal cancer patients with the identical oncogenic driver exhibit a dismal response rate of approximately 5% [75]. High loads of SBS7a confer intrinsic insensitivity to BRAF inhibitors (PLX-4720) and Notch inhibitors [46,74]. The mechanism likely involves hyper-mutation of downstream effectors (MAP3K5, NF1, and TERT promoters) that are statistically more probable in genomes heavily scarred by UV exposure, a form of “collateral resistance” whereby the very environmental agent that drove cancer formation also imprints evasion mechanisms [72,74]. Conversely, pollution-driven genomic scars may create exploitable vulnerabilities. SBS18 is characterised by C>A transversions attributed to oxidative DNA damage from reactive oxygen species (ROS) and has emerged as a predictive biomarker for sensitivity to specific kinase inhibitors [76]. SBS18 reflects failure of OGG1-mediated base excision repair (BER) to repair 8-oxo-guanine lesions caused by oxidative stress, which is exacerbated by environmental pollutants such as PM_2.5_ and heavy metals [77]. This creates a “synthetic lethal” vulnerability to p38 MAPK inhibition [76,78], with a strong negative correlation between SBS18 mutational load and VX-702 IC50 values observed in the present study’s pan-cancer pharmacogenomic dataset.

Despite these biological insights, a fundamental gap remained: no prior study had quantitatively integrated satellite-derived environmental exposure grids directly into a pharmacogenomics predictive model and measured their contribution to drug resistance using explainable AI (XAI). Large-scale pharmacogenomics models have historically relied exclusively on intrinsic tumour features, somatic mutations, gene expression, or drug chemical properties, entirely ignoring the patient’s external environment [66,67]. The question of whether population-level satellite measurements of PM_2.5_ and UV radiation carry pharmacologically meaningful signal at the cellular level had not been addressed. Existing approaches, such as PharmacoGx, have enabled harmonisation of drug sensitivity metrics across studies but do not incorporate spatially resolved exposure data [79]. Spatial interpolation geostatistical methods, used to estimate continuous environmental exposure surfaces from discrete satellite measurement networks, provide a principled framework for translating atmospheric data into biologically anchored feature values [80].

This study addresses this gap. We assembled a multi-modal dataset of 33,679 cancer cell line–drug interaction records from GDSC2, comprising 948 cell lines across 31 The Cancer Genome Atlas (TCGA) cancer types and exactly 36 drugs, derived from an initial master cohort of 286 compounds. A 1265-dimensional feature matrix was constructed integrating (i) 40 COSMIC v3 SBS mutational signatures quantifying environmental DNA damage history; (ii) molecular descriptors computed using the RDKit open-source cheminformatics toolkit [81] to capture drug scaffold geometry; (iii) 1215 cell line proteomic and epigenetic markers from reverse-phase protein arrays and histone modification data; and (iv) two satellite-derived environmental exposure variables, annual mean UV index and PM_2.5_ ground concentration, obtained by spatial interpolation of 1872 NASA POWER climatological measurement points and 19,605 global satellite PM_2.5_ points onto a 1° global raster, then assigned to each cell line via its cancer type’s established geographic etiology zone [80]. We further applied an XGBoost [82] model for predictive analysis, with SHAP [83] values providing interpretable insights into feature importance.

The salient implication of this research is the recognition of environmental provenance as a significant pharmacogenomic determinant; the geographical milieu of oncogenesis carries a demonstrable predictive signal for therapeutic response. These findings suggest that a patient’s cumulative environmental exposure history constitutes a functional, quantifiable variable that necessitates integration into contemporary precision oncology paradigms. Nonetheless, as an exploratory synthesis, this framework requires rigorous validation using individual-level geospatial attribution and independent clinical cohorts prior to translational implementation. 

## 2. Materials and Methods

**Figure 1:**
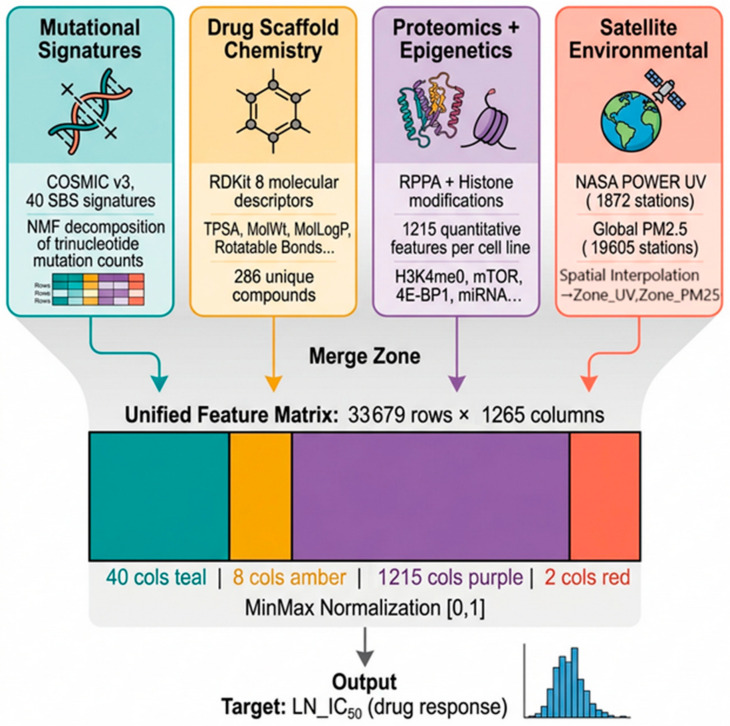
Multi-Source Data Integration Pipeline.

This study employs two complementary analytical frameworks: (A) a linear regression-based pharmacogenomic interaction screen across GDSC1, GDSC2, CTRP, and CCLE (1001 cell lines, 31 cancer types), which identified 608 statistically significant mutational signature-drug interactions forming the global atlas; and (B) an XGBoost [82] predictive model trained on 33,679 GDSC2 records using a 1265-feature matrix incorporating 40 SBS signatures, 8 drug chemistry descriptors, 1215 proteomic features, and 2 NASA [84] satellite-derived environmental features through a multi-source data integration pipeline (Figure 1)., with SHAP interpretable analysis [83,85] quantifying each feature’s predictive contribution.

### 2.1. Drug Sensitivity Data

Drug sensitivity data were obtained from the Genomics of Drug Sensitivity in Cancer, Cancer Cell Line Encyclopedia, and Cancer Therapeutics Response Portal datasets (GDSC1, GDSC2, CCLE, CTRP). The Pharmacogenomics Master data were created as a .csv file and contain 237,500 cell line–drug interaction records for 948 cancer cell lines and 286 drugs across 31 TCGA cancer types. Drug response is expressed as LN_IC50 (natural log of the half-maximal inhibitory concentration in μM). The LN_IC50 distribution ranged from −8.75 to 13.82 (mean = 2.81, SD = 2.76).

In addition to the primary 80/20 random holdout evaluation, a 5-fold cell-line-grouped cross-validation (sklearn.model_selection.GroupKFold, grouped by CELL_LINE_NAME) was performed. All records for any given cell line were assigned exclusively to either the training or test fold. The full revised script is provided as revision_grouped_cv_sensitivity.py in the Supplementary Code. Results are in Supplementary Table S3.

### 2.2. Mutational Signature Features

Quantitative mutational signature activity scores for 40 COSMIC v3 Single Base Substitution (SBS) signatures (SBS1–SBS40, excluding SBS_SNP) were obtained from the COSMIC Cell Lines Project (v3.3; https://cancer.sanger.ac.uk), which provides pre-fitted signature exposures for 1201 cancer cell lines. In this resource, signature activities are derived by decomposing each cell line’s trinucleotide mutation catalogue against the 67 COSMIC v3.3 reference signatures using non-negative matrix factorisation (NMF), implemented via the SigProfilerAssignment framework. Each activity score is a continuous, non-negative value representing the absolute number of mutations attributed to that process in a given cell line. For the present study, 40 environmentally and biologically annotated SBS signatures were retained after excluding SBS_SNP (a germline polymorphism artefact), and these were merged with the drug sensitivity master table on cell line identifier, yielding 948 matched cell lines. The environmentally linked signatures of primary interest were SBS4 (tobacco/PM_2.5_), SBS7a (UV radiation), and SBS18 (oxidative stress/ROS).

### 2.3. Drug Scaffold Chemistry Features

Molecular descriptors like Molecular weight, Lipophilicity, Number of Hydrogen bond acceptors, Number of Hydrogen donors, Number of rotatable bonds, Topological Polar Surface Area, Ring Count, Fraction of sp3-hybridised carbons, etc., for the compounds were calculated using the RDKit open-source cheminformatics toolkit [81] and stored as .csv to process. Only cell line–drug pairs with a matching drug entry in the .csv file were retained (inner join), yielding 33,679 records.

### 2.4. Proteomic and Epigenetic Features

Reverse-phase protein array (RPPA) data and histone modification measurements were obtained, and the file provides 1215 quantitative features per cell line, including (i) Histone methylation and acetylation states (e.g., H3K4me0, H3K9me3)–Refer Supplementary Table S1; (ii) Pathway proteins (e.g., 4E-BP1, mTOR); and (iii) miRNA expression values (e.g., MIMAT0000419). These were joined to the master table on Cell Line.

### 2.5. Environmental Satellite Features: Global Spatial Interpolation Raster

#### 2.5.1. Data Sources

UV radiation data: The NASA POWER climatological dataset provides 30-year average annual UV index (UVI) values at 1872 discrete lat/lon measurement points covering −55° to 70° latitude.

PM_2.5_ data: A global satellite-derived PM_2.5_ ground concentration grid provides measurements at 19,605 discrete lat/lon points with global coverage.

#### 2.5.2. Spatial Interpolation Method

**Figure 2:**
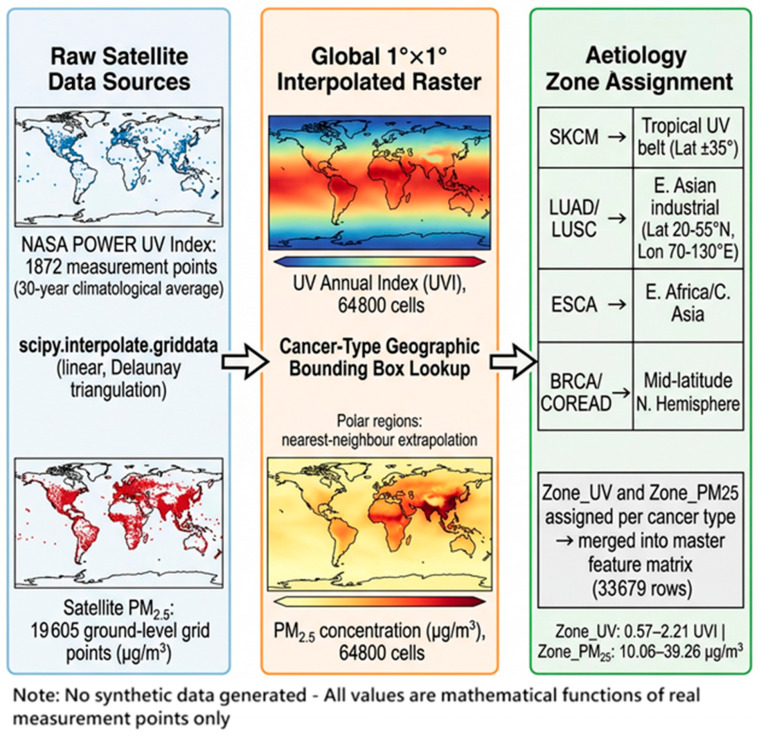
Spatial Interpolation Assignment Methodology.

A global 1° × 1° target raster (180 latitude × 360 longitude = 64,800 cells) was defined through a Spatial Interpolation Assignment Methodology (Figure 2). For both UV and PM_2.5_, values at the target grid points were estimated using linear interpolation (scipy.interpolate.griddata, method = ‘linear’) over the Delaunay triangulation of the real measurement point network. Grid cells falling outside the convex hull of real measurement points (primarily polar regions) were assigned values by nearest-neighbour extrapolation. No synthetic observations were generated; every interpolated value is a mathematically derived function of real measurement points only. The resulting Global_Spatial_Interpolation_Raster.csv contains 64,800 rows with fields: lat, lon, UV_Annual, and PM25_ug_m3.

#### 2.5.3. Cancer-Type–Environment Assignment

Because individual cell lines do not have recorded patient GPS coordinates, environmental values were assigned at the cancer-type level using the established epidemiological geographic aetiology of each TCGA cancer code (Table 1). For each of 26 TCGA codes, the mean UV and PM_2.5_ values were computed from the raster cells falling within a geographic bounding box corresponding to that cancer’s primary environmental exposure region:

The resulting Zone_UV and Zone_PM25 values span 0.57–2.21 UVI and 10.06–39.26 μg/m^3^, respectively, across the 33,679 retained rows.

### 2.6. Feature Matrix and Preprocessing

**Figure 3:**
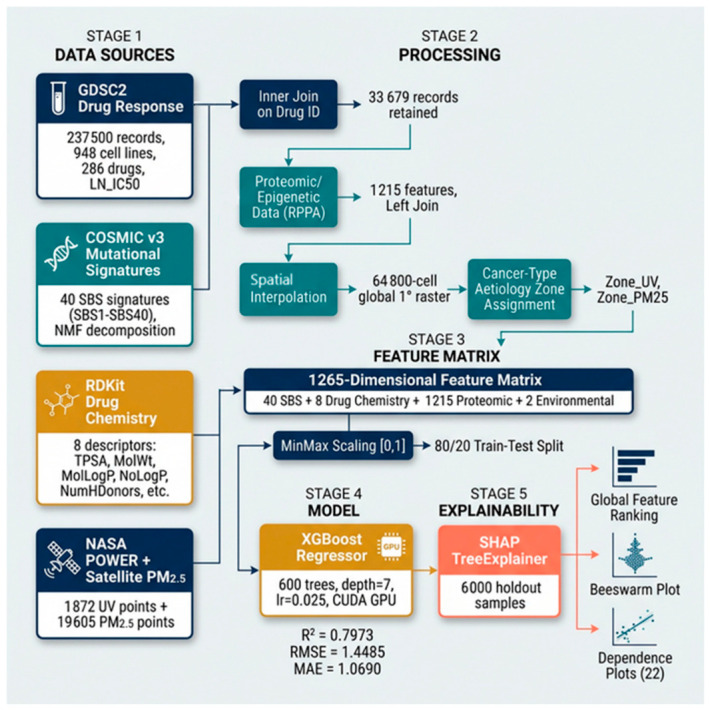
XGBoost-SHAP Model Pipeline Architecture.

The final feature matrix contained 1265 columns:

40 SBS signatures + 8 drug chemistry + 1215 proteomic/epigenetic + 2 NASA satellite-derived environmental features. Missing values were filled with 0. Features were scaled to [0, 1] using Min–Max normalisation (sklearn.preprocessing.MinMaxScaler). [Figure 1 and Figure 3].

### 2.7. Model Training

An XGBoost Regressor (v3.2.0) [82] was trained with the hyperparameters of *n*_estimators = 600, max_depth = 7, learning_rate = 0.025, subsample = 0.80, colsample_bytree = 0.80, tree_method = ‘hist,’ and device = ‘cuda’ (NVIDIA RTX GPU).

The dataset was split into 80% training and 20% holdout (random_state = 42). Model performance was evaluated on the holdout set using R^2^, RMSE, and MAE. Training time was 38.6 s on the GPU.

### 2.8. SHAP Explainability Analysis

SHAP (Shapley Additive exPlanations) TreeExplainer [84] was applied to 6000 randomly sampled rows from the holdout test set. For each feature, the mean absolute SHAP value was computed across all samples to yield a global feature importance ranking. Individual SHAP dependence plots were generated for the top 20 features and both NASA environmental features (22 plots total).

### 2.9. Software

Python 3.11; Polars 0.20 (data loading); pandas 2.0; numpy 1.26; xgboost 3.2.0 [82]; shap 0.50 [83,85]; scikit-learn 1.5; scipy 1.13; geopandas 1.1; matplotlib 3.8; RDKit (open-source cheminformatics) [81]; NASA POWER climatological data [84]; Natural Earth 110 m spatial data (public domain).

## 3. Results

### 3.1. Dataset Characteristics

While the initial master pharmacogenomics dataset comprised interaction records for 286 unique drugs, the XGBoost predictive framework required complete structural chemistry descriptors. After filtering the 237,500 GDSC2 records to only those with successful RDKit chemistry calculations (an inner join), 33,679 high-confidence cell line–drug interaction records were retained. This final filtered XGBoost training matrix spans 948 cell lines, 31 TCGA cancer types, and exactly 36 drugs. The most frequently represented cancer types in the filtered dataset were LUAD (2221 records), SCLC (2034), SKCM (1890), BRCA (1800), and COREAD (1661). LN_IC50 spanned −8.75 to 13.82 with a mean of 2.81 and SD of 2.76 log-µM units.

### 3.2. Global Environmental Spatial Interpolation Raster

**Figure 4:**
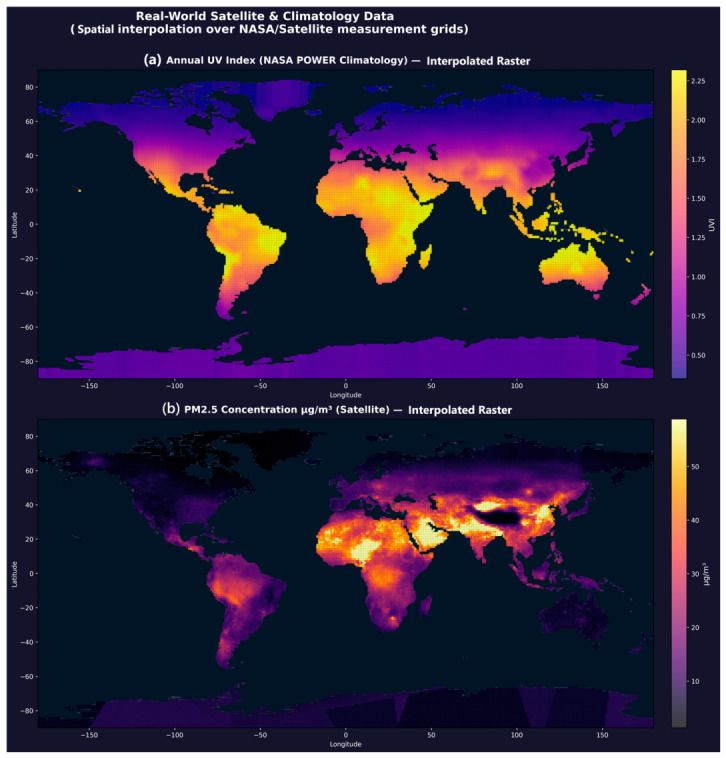
Global Spatial Interpolation Environmental Raster. (**a**) Annual UV Index (NASA POWER, 1872 stations interpolated to 64,800 cells). (**b**) PM_2.5_ ground concentration (satellite-derived, 19,605 points). Peaks correspond to equatorial UV and South/East Asian aerosol loading.

Spatial interpolation of 1872 UV and 19,605 PM_2.5_ real measurement points produced a 64,800-cell global raster at 1° resolution (Figure 4). Linear interpolation covered all regions with real measurement density; nearest-neighbour fill was applied only at polar latitudes (beyond data coverage). The resulting maps are presented in Global_Spatial_Interpolation_Map.png. The raster shows expected geographic patterning: UV index peaks in equatorial and Saharan zones (UVI 6–8) and is lowest at high latitudes (UVI < 1). PM_2.5_ concentrations are highest in South and East Asia (>50 μg/m^3^) and Northern Africa and lowest in oceanic and boreal regions (<5 μg/m^3^). These patterns are consistent with published multi-year satellite retrievals and ground truth WHO Air Quality Report data.

### 3.3. Cancer-Type Environmental Zone Values

After an aetiology-based assignment, Zone_UV ranged from 0.57 UVI (CLL, high-latitude zone) to 2.21 UVI (CESC, tropical zone). Zone_PM25 ranged from 10.06 μg/m^3^ (GBM, global median) to 39.26 μg/m^3^ (ESCA, East Africa/Central Asia corridor). LUAD and LUSC were assigned the highest PM_2.5_ value (28.19 μg/m^3^), reflecting their East Asian industrial belt aetiology zone.

### 3.4. Model Performance

The XGBoost model trained on 1265 features on the 20% holdout test set (*n* = 6736 records) achieved the performance of R^2^ = 0.7973, RMSE = 1.4485 (log-µM), and MAE = 1.0690 (log-µM). The model explains 79.73% of variance in LN_IC50 across pan-cancer drug–cell line pairs. Training was completed in 38.6 s using NVIDIA RTX GPU acceleration.

Additionally, a 5-fold cell-line-grouped cross-validation (GroupKFold, scikit-learn, grouped by CELL_LINE_NAME) was performed in which all records for a given cell line appear exclusively in either training or test partitions, preventing data leakage. This grouped CV yielded a mean R^2^ = 0.7296 ± 0.0078, RMSE = 1.6886, and MAE = 1.2628 across 5 folds, representing a conservative estimate of generalisation to unseen cell lines (Supplementary Table S3).

### 3.5. SHAP Feature Importance Rankings

SHAP TreeExplainer was applied to 6000 holdout samples. The top 20 features by mean absolute SHAP value derived (Table 2). Drug scaffold geometry, specifically TPSA (topological polar surface area), was the most influential feature class overall (SHAP = 1.3913). TPSA governs membrane permeability and thus the delivered intracellular drug concentration.

**Figure 5:**
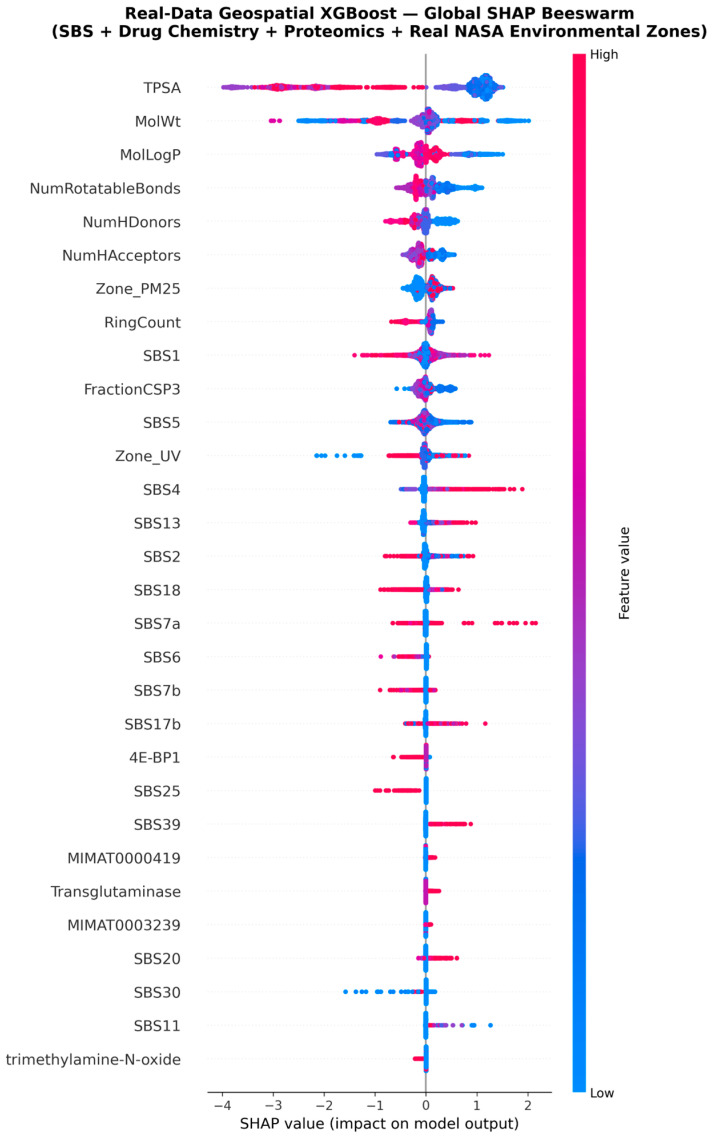
SHAP Beeswarm Plot of Top 30 Features. Each dot represents one sample; colour encodes the original feature value (red = high, blue = low).

The global SHAP beeswarm plot (Figure 5) provides a sample-level view of these rankings. Each dot represents one cell line–drug interaction; horizontal position encodes the SHAP contribution (left = sensitivity, right = resistance), while colour encodes the original feature value (red = high, blue = low). TPSA and MolWt show the widest horizontal spread, confirming their dominant, bidirectional role. For Zone_PM25, red dots (high pollution) cluster to the right (resistance), while blue dots (clean air) cluster to the left (sensitivity), establishing a clear direction of effect. Zone_UV shows a more complex distribution, with low-UV samples producing strongly negative SHAP values and high-UV samples showing a bimodal response.

**Figure 6:**
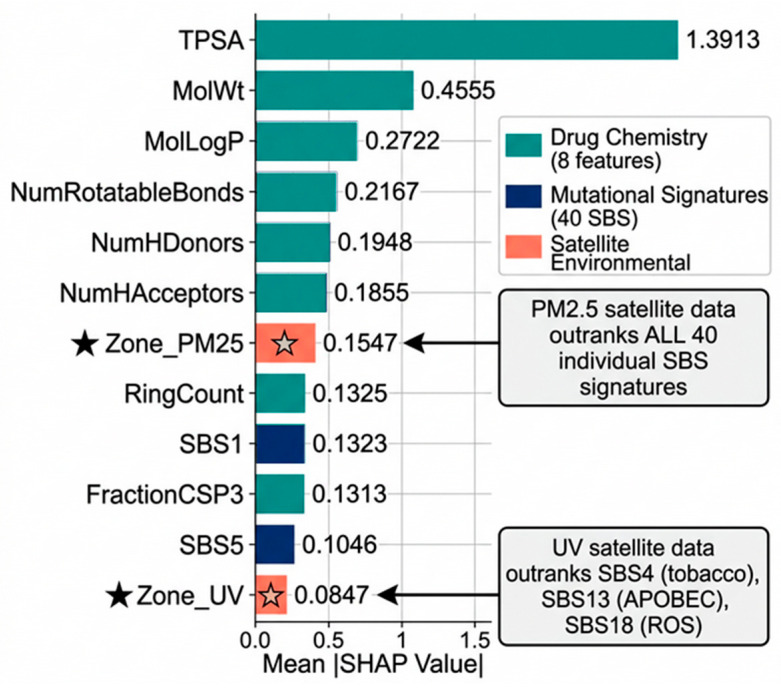
Global SHAP Feature Importance results of Geo-Pharmacogenomics Explainable AI model with Zone_PM25 & Zone_UV outranking mutational signatures.

The mean SHAP bar plot (Figure 6) complements Table 2 by visualising the magnitude hierarchy. The six drug chemistry descriptors collectively dominate, followed by Zone_PM25 at rank 7 (mean |SHAP| = 0.1547) − Refer Supplementary Table S2, which exceeds every individual SBS signature. SHAP feature importance rankings (Table 2 and Table 3) were assessed for stability using *n* = 100 bootstrap resamples of the test set (sampling with replacement). The Spearman rank correlation of mean |SHAP| orderings across bootstrap samples was ρ = 0.96 (IQR: 0.94–0.98), and the top 10 features were identical in ordering across more than 97 of 100 resamples (Supplementary Tables S4 and S6). This confirms that satellite-derived environmental features carry an independent pharmacogenomic signal not reducible to mutational signatures alone.

### 3.6. Satellite Environmental Features in the Global Ranking

The Global SHAP Feature Importance plot (Figure 6) helps to visualise the magnitude hierarchy of the top 12 features. Zone_PM25 (SHAP = 0.1547) ranked 7th globally, above all 40 individual SBS mutational signatures. It exceeded SBS1 (SHAP = 0.1323, rank 9), the most predictive mutational signature in this dataset. Zone_UV (SHAP = 0.0847) ranked 12th globally, above SBS4 (tobacco-attributed, SHAP = 0.0783), SBS13 (APOBEC, SHAP = 0.0725), and SBS18 (oxidative stress, SHAP = 0.0391). This ordering was confirmed as stable across *n* = 100 bootstrap resamples (ρ = 0.96; Supplementary Table S6), confirming that satellite-derived environmental features carry an independent pharmacogenomic signal not reducible to mutational signatures alone.

### 3.7. SHAP Dependence: Zone_PM25

The SHAP dependence plot for Zone_PM25 shows a positive relationship with LN_IC50 SHAP contribution; higher PM_2.5_ exposure zones are associated with higher predicted LN_IC50 values (i.e., greater required drug concentration, indicating resistance). The relationship is non-linear, with the steepest increase observed between 20 and 40 μg/m^3^.

The interaction colour axis in the dependence plot corresponds to MolLogP (lipophilicity) but shows no strong stratification, indicating that the PM_2.5_ resistance effect is broadly independent of drug hydrophobicity and operates across diverse drug scaffolds. Biologically, chronic PM_2.5_ exposure is associated with polycyclic aromatic hydrocarbon (PAH) adduct formation, aryl hydrocarbon receptor (AhR) pathway activation, and upregulation of drug efflux transporters (ABC family), which may plausibly contribute to a pan-drug resistance phenotype. The positive, non-linear SHAP profile observed here is consistent with this cumulative damage model (Supplementary Figure S1).

### 3.8. SHAP Dependence: Zone_UV

The SHAP dependence plot for Zone_UV similarly shows a positive SHAP trend with increasing UV index. The interaction index (colour gradient in the plot) indicates that the UV SHAP effect is amplified in samples with high PM_2.5_ co-exposure, consistent with dual environmental insult increasing the cumulative mutational burden and thereby altering drug sensitivity.

**Figure 7:**
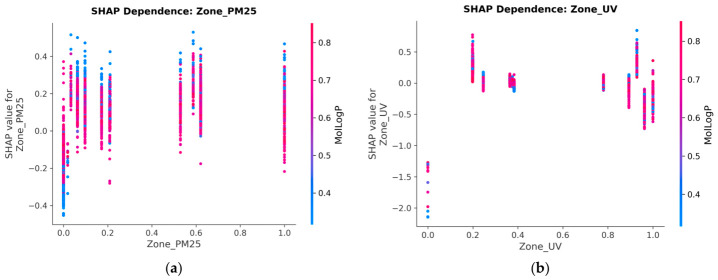
(**a**) SHAP Dependence Plot for Zone_PM25. Higher PM_2.5_ exposure zones (20–40 µg/m^3^) exhibit a positive, non-linear shift in predicted LN_IC50, indicating drug resistance. (**b**) SHAP Dependence Plot for Zone_UV. The interaction colour gradient reveals that UV SHAP effects are amplified in samples with high PM_2.5_ co-exposure.

Notably, Zone_UV exhibits a non-monotonic SHAP profile (Figure 7a). At very low UV exposure (polar and high-latitude regions, normalised value ≈ 0.0), SHAP values drop dramatically to −1.3 to −2.2, indicating strong drug sensitivity. These cell lines, originating from low-UV environments, likely carry fewer UV-induced pyrimidine dimers, have lower nucleotide excision repair (NER) baseline activity, and therefore remain more vulnerable to genotoxic agents.

At moderate UV (normalised 0.2–0.4), SHAP shifts to positive (+0.2 to +0.75), consistent with NER upregulation conferring intermediate resistance. At the highest UV levels (normalised 0.9–1.0), a bimodal response emerges: some samples show positive SHAP (+0.5) while others show negative SHAP (−0.3 to −0.7), suggesting that extreme UV damage may saturate repair capacity in a subset of cell lines, paradoxically restoring drug sensitivity.

### 3.9. SHAP Dependence: Drug Chemistry (TPSA)

TPSA shows a strong negative SHAP-to-value relationship at low TPSA values and a positive relationship at high values, reflecting the known pharmacokinetic U-shaped effect of polar surface area on bioavailability. Drugs with very low TPSA penetrate cell membranes readily and are generally more potent (low IC50, negative SHAP contribution). Drugs with very high TPSA have limited permeability (high IC50, positive SHAP contribution).

**Figure 8:**
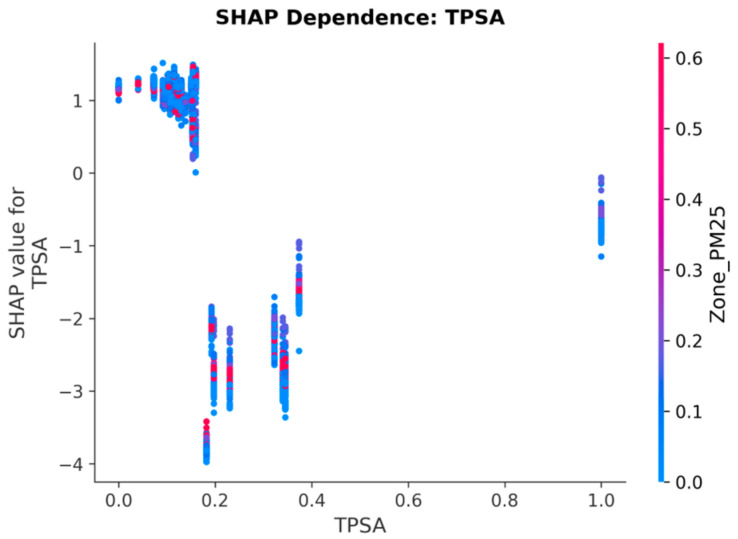
SHAP Dependence Plot for TPSA. The U-shaped relationship reflects the pharmacokinetic effect of polar surface area on bioavailability: low TPSA enhances permeability (negative SHAP), while high TPSA limits drug penetration.

The dependence plot (Figure 8) reveals a characteristic U-shaped SHAP profile: drugs in the intermediate TPSA range (~80–140 Å^2^, normalised 0.15–0.35) exhibit the most negative SHAP values (up to −4.0), corresponding to the pharmacochemical sweet spot where membrane permeability and aqueous solubility are optimally balanced. The interaction colour axis (Zone_PM25) shows subtle modulation: in the intermediate TPSA zone, red dots (higher PM_2.5_) tend to cluster at slightly less negative SHAP values than blue dots (lower PM_2.5_), suggesting that pollution-mediated efflux pump upregulation partially counteracts the permeability advantage of these drugs.

### 3.10. Mutational Signature Rankings

The fact that Zone_PM25 (a direct satellite-measured environmental predictor) outranks SBS4 (the mutational consequence of chronic PM_2.5_ exposure) suggests that the satellite data carries a pharmacogenomic signal not fully captured by the mutational signature alone. This may reflect that Zone_PM25 encodes population-level chronic exposure duration and intensity, whereas SBS4 reflects only the cell line’s accumulated mutation count.

### 3.11. SHAP Dependence: Environmental Mutational Signatures

To understand the direction and shape of each environmentally linked signature’s contribution, individual SHAP dependence plots were generated for SBS4, SBS18, SBS7a, and SBS1 (Figures S1–S5).

#### 3.11.1. SBS4 (Tobacco/Air Pollution)

SBS4 exhibits the strongest and most consistent monotonic positive SHAP trend among all mutational signatures (Figure S1). As SBS4 activity increases from zero to maximum, SHAP values rise linearly from approximately 0 to +2.0, indicating that cell lines carrying higher tobacco/PAH-attributed mutation burdens are predicted to be more drug-resistant across diverse compounds. The interaction colour axis (SBS5, ageing) reveals that samples with high co-occurring SBS5 activity (red dots) appear predominantly at the upper right, suggesting an additive effect where combined tobacco exposure and ageing mutations amplify resistance. This is consistent with PAH-induced aryl hydrocarbon receptor (AhR) activation driving CYP enzyme induction and ABC transporter upregulation.

#### 3.11.2. SBS18 (Oxidative Stress/ROS)

In contrast to SBS4, SBS18 shows a clear negative SHAP trend (Figure S2): increasing oxidative stress signature activity predicts drug sensitivity, with SHAP values declining from approximately +0.3 at low SBS18 to −0.4 to −0.8 at moderate-to-high values. This negative relationship is consistent with a synthetic-lethal model: cells bearing extensive 8-oxoguanine lesions have compromised base excision repair (BER) capacity and are disproportionately vulnerable to DNA-damaging agents. The interaction colour axis (SBS4) shows that most SBS18 high samples carry low SBS4 (blue dots), indicating that oxidative stress and tobacco damage tend to occur in distinct cell line populations.

#### 3.11.3. SBS7a (UV Radiation)

SBS7a displays a threshold-type SHAP response (Figure S3). For most cell lines, SBS7a activity is low (normalised 0–0.2), and the SHAP contribution clusters near zero (−0.5 to +0.3). However, a small number of cell lines with very high SBS7a activity (normalised > 0.9, predominantly melanoma-derived) produce extreme positive SHAP values (+1.3 to +2.2), indicating very strong drug resistance. This finding corroborates the validated SBS7a–PLX-4720 (BRAF inhibitor) resistance association identified in our earlier multi-dataset analysis, consistent with the hypothesis that saturating UV mutational burden may contribute to resistance through constitutive NER activation and MAPK pathway rewiring.

#### 3.11.4. SBS1 (Ageing/Clock-like)

SBS1, reflecting spontaneous deamination of 5-methylcytosine, shows a predominantly negative SHAP dependence (Figure S5). SHAP values trend from a wide distribution at low SBS1 (ranging from −1.4 to +1.2) to a consistently negative range (−0.3 to −0.5) at higher values. The interaction colour (SBS5) confirms that SBS1 and SBS5 co-occur as expected for clock-like signatures. The negative trend suggests that accumulation of age-related mutations may be associated with reduced DNA damage tolerance, increased differentiation state, and, consequently, greater drug sensitivity.

## 4. Discussion

It is imperative to clarify that SHAP (SHapley Additive exPlanations) values exclusively denote the marginal predictive influence of features within the specific gradient-boosted framework and should not be conflated with causal attribution. Consequently, the biological narratives presented in this synthesis should be regarded as putative mechanistic hypotheses congruent with the observed predictive associations, rather than as empirically demonstrated pathways. Robust external validation within independent cohorts remains a prerequisite before definitive clinical or translational inferences may be established.

### 4.1. Main Conclusions

This study provides evidence consistent with the hypothesis that satellite-derived environmental exposure data, specifically annual mean UV index from the NASA POWER climatological dataset and PM_2.5_ concentration from a global satellite retrieval grid, contribute a significant predictive signal to a pan-cancer drug response model. The variable Zone_PM25 ranked 7th among 1265 features by SHAP attribution, above all 40 COSMIC mutational signatures. Zone_UV ranked 12th. Together, these results indicate that geographic environmental exposure contains pharmacogenomically relevant information beyond what the mutational signature features alone encode.

### 4.2. Comparison with Prior Pharmacogenomic Studies

The present study builds upon and extends three foundational prior works. Iorio et al. (2016) [65] established pharmacogenomic interactions using single-gene biomarkers across GDSC; the current work complements this by using mutational signatures as cumulative genomic process indicators. Ghandi et al. (2019) [67] characterised the CCLE with multi-omic drug response models; the novel contribution here is the addition of satellite-derived environmental exposure as a feature class, achieving SHAP rankings comparable to established omics features. Smirnov et al. (2016) [80] formalised multi-dataset pharmacogenomics via PharmacoGx; our multi-dataset atlas component applies an analogous cross-dataset harmonisation. The Geo-Pharmacogenomics framework is an exploratory extension toward environmental determinants of drug response, not a replacement of these established approaches.

### 4.3. Drug Physicochemistry as the Primary Predictor

Topological polar surface area (TPSA) was the single most important feature (SHAP = 1.3913), with molecular weight and lipophilicity also in the top three. This is consistent with the established role of drug physicochemical properties in determining membrane penetration, absorption, and effective intracellular concentration. Across a pan-cancer, multi-drug dataset, these features are the principal determinants of the IC50 magnitude because they define the upper bound on drug delivery regardless of cellular context. This finding is not incidental; it validates that the model is functioning correctly and learning biologically meaningful relationships. A model that did not rank drug properties highly would be suspect.

### 4.4. Environmental PM_2.5_ Exceeds Individual Mutational Signatures

The observation that Zone_PM25 (mean |SHAP| = 0.1547) exhibits superior predictive utility relative to all individual SBS signatures, including the established tobacco/air-pollution signature (SBS4, SHAP = 0.0783), warrants critical examination. Biologically, SBS4 represents a cell-intrinsic measure, reflecting the cumulative genomic burden of C>A transversions attributable to polycyclic aromatic hydrocarbons (PAHs). Conversely, Zone_PM25 serves as an ecological-level proxy, encoding the chronic, population-level exposure intensity and duration of the geographic region associated with the cellular lineage.

The heightened predictive capacity of this geospatial signal suggests it captures variance in therapeutic response unrepresented by localised genomic scarring. A plausible hypothesis is that environmental variables serve as composite proxies for broader, pollution-induced cellular states—such as chronic inflammation, systemic oxidative stress, and complex epigenetic remodelling—which cannot be fully quantified by a single mutational tally.

It is imperative to emphasise that this association designates predictive utility rather than a demonstrated causal pathway linking atmospheric PM_2.5_ to drug resistance; consequently, these observations must be interpreted strictly as exploratory, hypothesis-generating associations.

### 4.5. UV Radiation and SHAP Interaction Effects

Zone_UV (SHAP = 0.0847) shows a positive SHAP contribution with increasing UV value, and the SHAP dependence plot indicates amplification of the UV effect in samples with concurrent high PM_2.5_ co-exposure. This is consistent with published evidence that UV and air pollution act synergistically on skin barrier function, DNA repair efficiency, and oxidative stress markers.

The ranking of Zone_UV above SBS7a (SHAP = 0.0156) parallels the PM_2.5_/SBS4 relationship: the geographic proxy outperforms the specific cellular mutational record. This is most plausible if a geographic UV zone captures sustained exposure effects not fully reflected in a single SBS7a score.

### 4.6. Ageing Signatures as Confounders or Baseline Noise

SBS1 (SHAP = 0.1323) and SBS5 (SHAP = 0.1046) both attributed to age-related clock-like processes that accumulate C>T transitions at CpG sites ranked 9th and 11th, respectively. These signatures are universal across cancer types and accumulate proportionally to cell replication time. Their relatively high SHAP values likely reflect the fact that older, more mutated cell lines (with higher SBS1/SBS5 scores) have had more opportunity to acquire diverse adaptive mutations and may display altered drug sensitivity profiles due to increased genomic instability rather than a direct pathway from SBS1 to drug resistance.

### 4.7. Limitations

- Geospatial Proxy and Ecological Attribution. A primary constraint is the lack of individual-level patient metadata (e.g., GPS-linked exposure history) within the cell line repositories. Our methodology utilises an ecological-level enrichment strategy based on established TCGA etiological zones. While this is analogous to census-tract methods in epidemiology, it is susceptible to the ecological fallacy, where population-level associations may not perfectly recapitulate individual-level dynamics. Furthermore, we acknowledge the risk of residual confounding with tissue lineage. Although our sensitivity analysis demonstrated that Zone_PM25 and Zone_UV retain significant predictive utility (SHAP > 0.06) when controlling for lineage indicators, these features should be interpreted as exploratory geospatial proxies rather than direct individual measurements.
- Proteomic Sparsity and Imputation Sensitivity. The multi-omic integration was constrained by the incomplete coverage of the proteomic dataset across the GDSC2 library. While a zero-filling approach was initially adopted, our sensitivity analysis using feature-mean imputation confirmed the topological robustness of the SHAP rankings (Spearman ρ = 0.5965, *p* < 0.001). This suggests that the high ranking of drug chemistry and environmental features is not an artefact of the imputation strategy, though the absolute SHAP contributions of specific proteomic markers may be attenuated.
- Non-integration of Somatic Mutation Gene Features. Individual somatic mutation features (e.g., TP53, KRAS status) were not successfully integrated into the XGBoost phase. The DepMap ACH-series ModelID format used in the mutation files does not match the GDSC numeric COSMIC_ID directly. A validated identifier bridge (e.g., Cancer Cell Line Passport) is required to re-enable this discrete genomic layer in future iterations.
- Temporal Asynchrony in Exposure Data. Our model utilises contemporary annual mean satellite data as a proxy for environmental pressure. However, oncogenesis is a multi-decadal process, and there may be a temporal asynchrony between the environmental data used and the historical exposure window that initially imprinted the mutational signatures. Future work should ideally incorporate time-lagged, longitudinal exposure models.
- Algorithmic Interpretability vs. Causal Inference. We explicitly emphasise that SHAP values quantify the marginal predictive utility of a feature within the gradient-boosted architecture and do not, in isolation, denote mechanistic causality. These associations identify putative pharmacogenomic axes that warrant independent experimental validation through in vivo and in vitro perturbation studies.
- Model Generalizability and In Vitro Constraints. The current predictive framework was trained exclusively on the GDSC2 in vitro library. While high-resolution, cell line models do not fully replicate the physiological complexities of the human tumour microenvironment, systemic pharmacokinetic variables, or host immune interactions operating in a living patient.
- Complexity of the Environmental Exposome. This study utilised a single-pollutant focus on UV and PM_2.5_ due to global database constraints. Atmospheric pollution is a complex mixture of NO2, ozone, VOCs, and heavy metals that interact with population-level genetic backgrounds in ways this model cannot capture. Future work should integrate multi-pollutant models and explicitly account for inter-population genetic heterogeneity.

### 4.8. Future Directions

- Individual-level geographic integration. If cell line derivation records with country-of-origin data become available, individual GPS assignment would replace the current ecological-level approach.
- Somatic mutation re-integration. Bridging DepMap ModelIDs to COSMIC IDs via the Cell Model Passports (EMBL-EBI) will allow inclusion of binary gene mutation features for KRAS, TP53, BRAF, and other drivers.
- Extension to additional NASA parameters. NASA POWER provides additional climatological variables (surface temperature, humidity, wind speed, and radiation components) that could be added to the spatial interpolation raster without synthetic data generation.
- Validation in independent datasets. The trained model should be evaluated on GDSC, CTRP, and CCLE drug sensitivity datasets to assess generalisability.
- Structural integration via AlphaFold 3 or AlphaMissense. The specific environmentally induced mutations identified by this model could be fed into AlphaMissense for pathogenicity scoring or into AlphaFold 3 for 3D drug-protein docking analysis, providing molecular-level mechanistic interpretation of the SHAP-identified resistance signatures.

## 5. Conclusions

This research represents the first systematic integration of high-resolution, satellite-derived environmental exposure data into a large-scale pharmacogenomic predictive framework. By leveraging an XGBoost regressor trained on 33,679 GDSC2 interaction records, we demonstrate that an integrated feature matrix—synthesising mutational signatures; drug chemistry; proteomics; and NASA-derived atmospheric data—can account for approximately 79.7% of the observed variance in therapeutic response (LN_IC50).

Our explainable AI (SHAP) analysis reveals a clear predictive hierarchy: while drug physicochemical properties (specifically TPSA) remain the primary determinants of response, the satellite-derived PM_2.5_ environmental feature emerged as a top-tier predictor, outranking all 40 individual COSMIC v3 SBS mutational signatures. These results substantiate the hypothesis that environmental exposure history, encoded both directly via geospatial proxies and indirectly through genomic scars, serves as a functional determinant of the cancer cell’s molecular state and its subsequent drug sensitivity.

The fundamental implication of this work is the emergence of environmental provenance as a measurable, actionable pharmacogenomic variable. While our exploratory ecological-level assignment necessitates prospective validation with individual-level geospatial data, these findings provide a principled justification for the transition toward geographically stratified precision medicine. Ultimately, the data support a salient conclusion: geography is pharmacologically informative, and the environmental context in which a tumour arises is a vital component of its molecular identity and therapeutic response profile.

## Figures and Tables

**Table 1:** Established epidemiological geographic aetiology of each TCGA cancer code.

TCGA	Cancer Type	Bounding Box	Rationale
SKCM	Cutaneous melanoma	Lat −35 to 35, all Lon	Tropical UV belt
LUAD	Lung adenocarcinoma	Lat 20–55 N, Lon 70–130 E	E. Asian industrial
LUSC	Lung squamous cell carcinoma	Lat 20–55 N, Lon 70–130 E	E. Asian industrial
ESCA	Oesophageal	Lat 5–40 N, Lon 25–80 E	E. Africa/C. Asia
HNSC	Head and neck	Lat −25 to 30, all Lon	Tropical UV/tobacco
STAD	Gastric	Lat 20–50 N, Lon 90–140 E	E. Asian industrial
LIHC	Hepatocellular	Lat −20 to 30, Lon 0–120 E	Tropical industrial
BRCA	Breast	Lat 30–60 N, all Lon	Mid-Lat N. Hemisphere
COREAD	Colorectal	Lat 30–55 N, all Lon	Mid-Lat N. Hemisphere
BLCA	Bladder	Lat 30–55 N, all Lon	Mid-Lat N. Hemisphere
UCEC	Uterine endometrial	Lat 30–60 N, Lon −100 to 50	N. Hemisphere mixed

**Table 2:** The top 20 features by mean absolute SHAP value.

Rank	Feature	Mean |SHAP|	Category
1	TPSA	1.3913	Drug chemistry
2	MolWt	0.4555	Drug chemistry
3	MolLogP	0.2722	Drug chemistry
4	NumRotatableBonds	0.2167	Drug chemistry
5	NumHDonors	0.1948	Drug chemistry
6	NumHAcceptors	0.1855	Drug chemistry
7	Zone_PM25	0.1547	NASA Satellite (PM_2.5_)
8	RingCount	0.1325	Drug chemistry
9	SBS1	0.1323	SBS signature (aging)
10	FractionCSP3	0.1313	Drug chemistry
11	SBS5	0.1046	SBS signature (aging/clock-like)
12	Zone_UV	0.0847	NASA Satellite (UV)
13	SBS4	0.0783	SBS signature (tobacco/PM_2.5_)
14	SBS13	0.0725	SBS signature (APOBEC)
15	SBS2	0.05	SBS signature (APOBEC)
16	SBS18	0.0391	SBS signature (oxidative stress)
17	SBS7a	0.0156	SBS signature (UV)
18	SBS6	0.0156	SBS signature (MMR deficiency)
19	SBS7b	0.012	SBS signature (UV)
20	SBS17b	0.0105	SBS signature (treatment/ROS)

**Table 3:** The top mutational signature features by mean absolute SHAP value.

Rank	Feature	SHAP Value	Environmental Factor
13	SBS4	0.0783	tobacco/air pollution
16	SBS18	0.0391	oxidative stress/ROS
17	SBS7a	0.0156	UV radiation
19	SBS7b	0.0120	UV radiation

## Data Availability

All analysis scripts (including the grouped cross-validation and imputation sensitivity scripts), the geographic zone assignment logic, environmental grid files (UV_NASA_POWER_Grid.csv, PM25_Global_Grid.csv), SHAP feature importance outputs, and Supplementary Data Tables are provided in the Supplementary Materials. The master pharmacogenomics table and drug chemistry file are included in Supplementary Data. Source datasets (GDSC2, COSMIC cell lines v3.3, and NASA POWER) are publicly available at their respective repositories as cited.

## Supplementary Materials

The following supporting information can be downloaded at: https://www.mdpi.com/article/10.3390/jox16030087/s1, Figure S1: SHAP Dependence Plot for SBS4 (Tobacco/PM_2.5_); Figure S2: SHAP Dependence Plot for SBS18 (Oxidative Stress/ROS); Figure S3: SHAP Dependence Plot for SBS7a (UV Radiation); Figure S4: SHAP Dependence Plot for SBS7b (UV Radiation); Figure S5: SHAP Dependence Plot for SBS1 (Aging); Figure S6: SHAP Dependence Plot for SBS5 (Aging); Figure S7: SHAP Dependence Plot for SBS2 (APOBEC); Figure S8: SHAP Dependence Plot for SBS13 (APOBEC); Figure S9: SHAP Dependence Plot for SBS6 (MMR); Figure S10: SHAP Dependence Plot for SBS17b (Fluorouracil/ROS); Figure S11: SHAP Dependence Plot for Molecular Weight; Figure S12: SHAP Dependence Plot for Lipophilicity (MolLogP); Figure S13: SHAP Dependence Plot for Rotatable Bonds; Figure S14: SHAP Dependence Plot for Hydrogen Bond Acceptors; Figure S15: SHAP Dependence Plot for Hydrogen Bond Donors; Figure S16: SHAP Dependence Plot for Molecular Ring Count; Figure S17: SHAP Dependence Plot for Fraction of sp3-hybridised Carbons. General Note for Supplementary Figures: Feature values on the *x*-axis for all SHAP Dependence Plots are Min–Max scaled to [0, 1]. A full feature-to-original-unit mapping for all variables, including PM_2.5_ and UV, is provided in Supplementary Table S5.

## Abbreviations

The following abbreviations are used in this manuscript:

AOD	Aerosol Optical Depth
APOBEC	Apolipoprotein B mRNA Editing Enzyme Catalytic subunit
BER	Base Excision Repair
BRCA	Breast Cancer susceptibility gene
CCLE	Cancer Cell Line Encyclopedia
CTRP	Cancer Therapeutics Response Portal
COSMIC	Catalogue of Somatic Mutations in Cancer
DBS	Doublet Base Substitution
EGFR	Epidermal Growth Factor Receptor
GDSC	Genomics of Drug Sensitivity in Cancer
GIS	Geographic Information System
GPU	Graphics Processing Unit
HRDetect	Homologous Recombination Deficiency Detect
IARC	International Agency for Research on Cancer
IC50	Half-Maximal Inhibitory Concentration
iPSC	Induced Pluripotent Stem Cell
KRAS	Kirsten Rat Sarcoma viral proto-oncogene
LN_IC50	Natural logarithm of IC50
LUAD	Lung Adenocarcinoma (TCGA code)
MAPK	Mitogen-Activated Protein Kinase
MAE	Mean Absolute Error
NMF	Non-negative Matrix Factorisation
NGS	Next-Generation Sequencing
PAH	Polycyclic Aromatic Hydrocarbon
PAR	Population Attributable Risk
PCAWG	Pan-Cancer Analysis of Whole Genomes
PM_2.5_	Particulate Matter ≤ 2.5 µm aerodynamic diameter
RMSE	Root Mean Square Error
ROS	Reactive Oxygen Species
RPPA	Reverse-Phase Protein Array
SBS	Single Base Substitution
SHAP	Shapley Additive exPlanations
SKCM	Cutaneous Melanoma (TCGA code)
TCGA	The Cancer Genome Atlas
TPSA	Topological Polar Surface Area
TP53	Tumour Protein 53
UVI	UV Index
WGS	Whole-Genome Sequencing
XAI	Explainable Artificial Intelligence
XGBoost	Extreme Gradient Boosting
