# Supplementary figures and images for: Global Geo-Pharmacogenomics: Environmental Mutational Signatures Drive Population-Level Heterogeneity in Anticancer Drug Response
Source: J Xenobiot. 2026 May 18;16(3):87. doi: 10.3390/jox16030087 (PMC13214943; doi:10.3390/jox16030087)

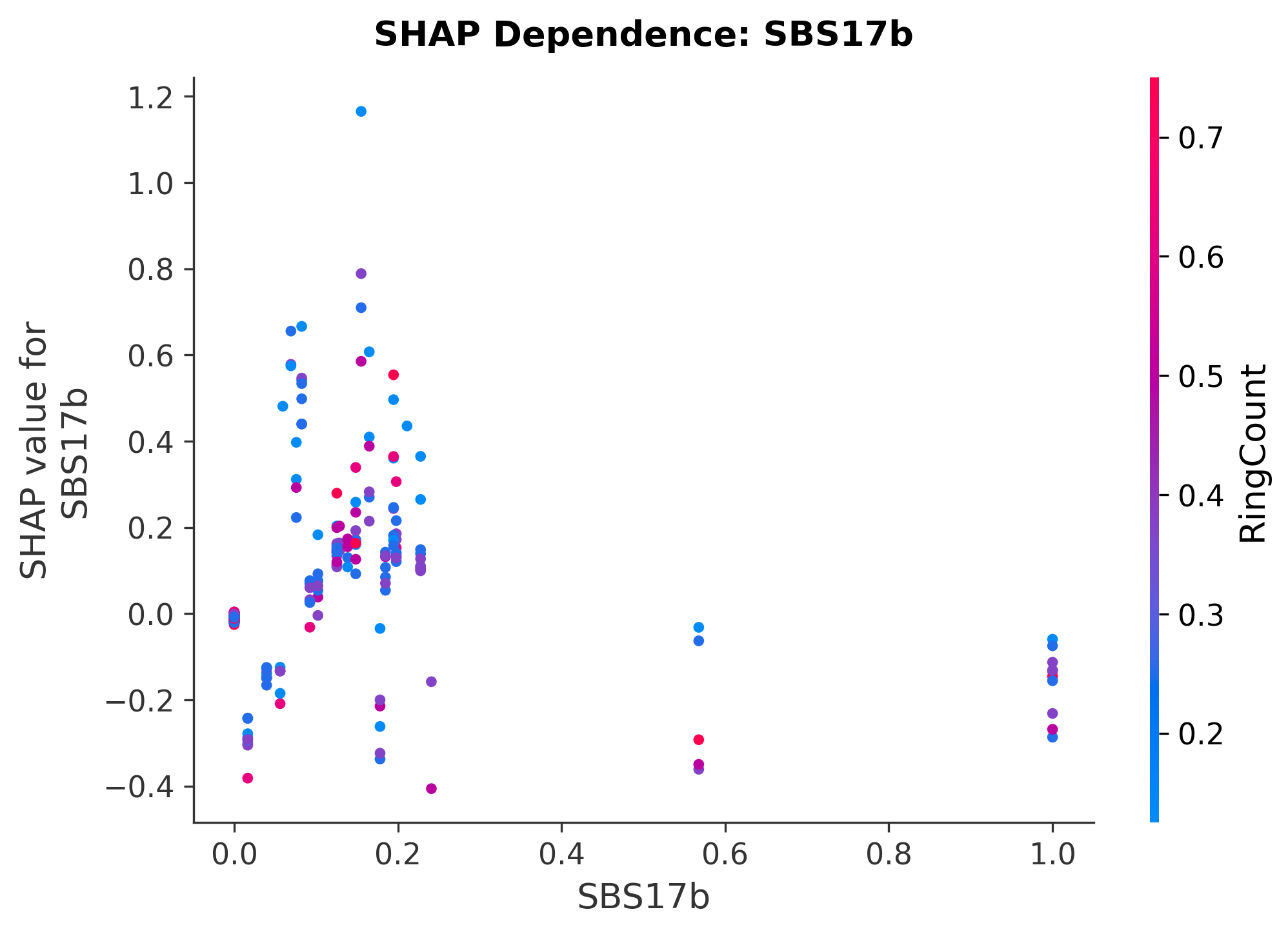

Supplement: Supplementary file 1 [file jox-16-00087-s001.zip › Supplementry_Figures/Figure_S10_SBS17b.png]

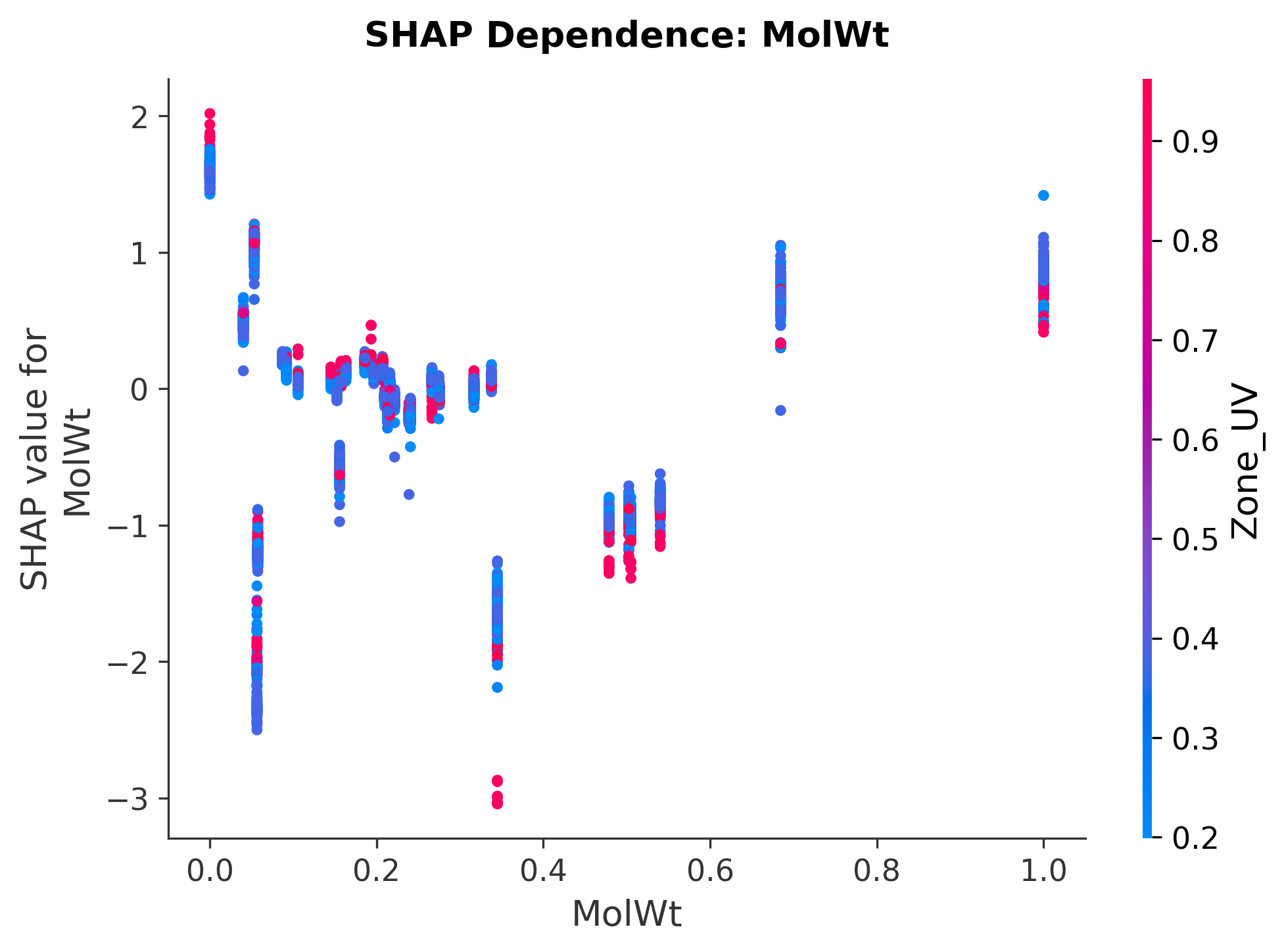

Supplement: Supplementary file 1 [file jox-16-00087-s001.zip › Supplementry_Figures/Figure_S11_MolWt.png]

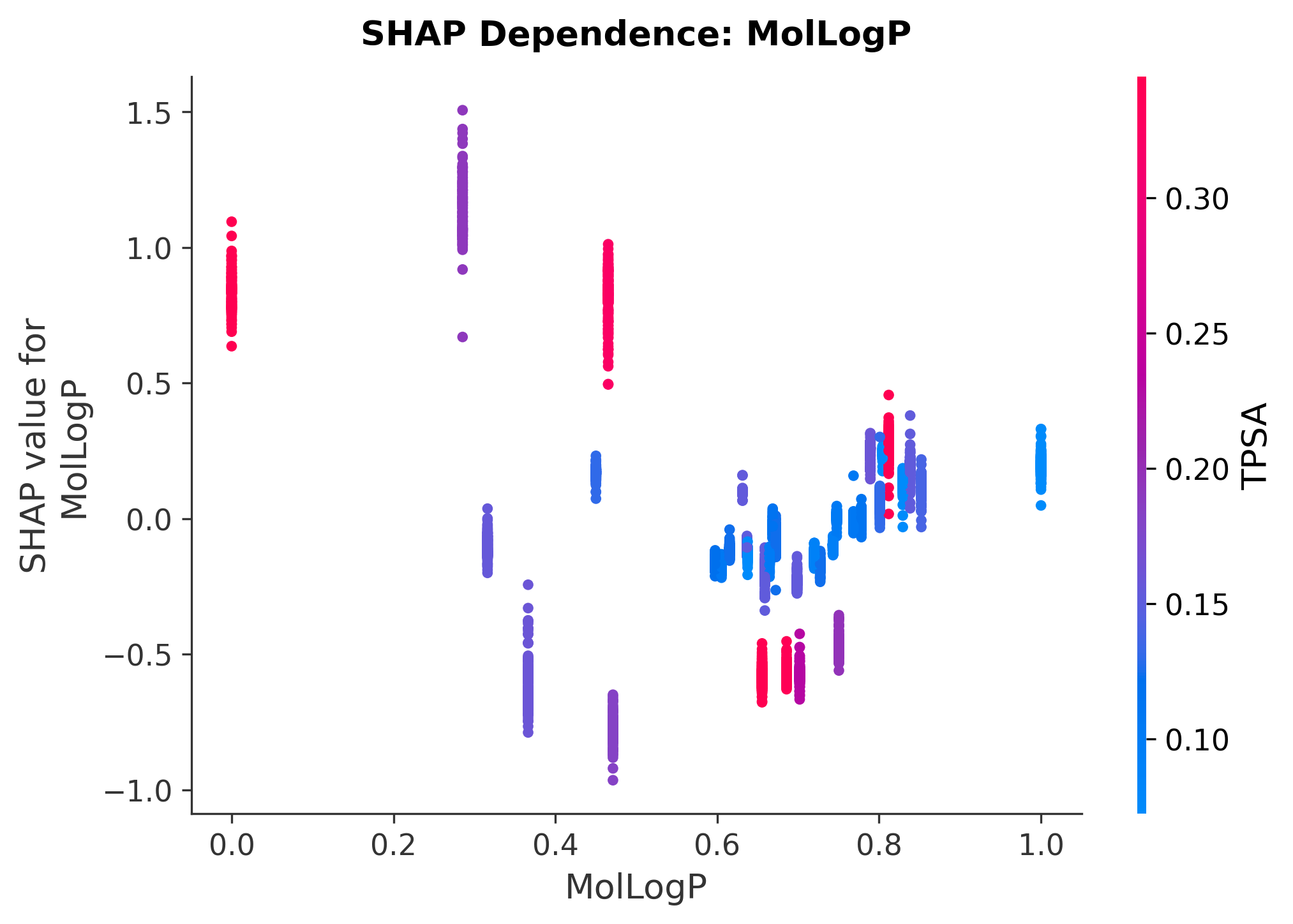

Supplement: Supplementary file 1 [file jox-16-00087-s001.zip › Supplementry_Figures/Figure_S12_MolLogP.png]

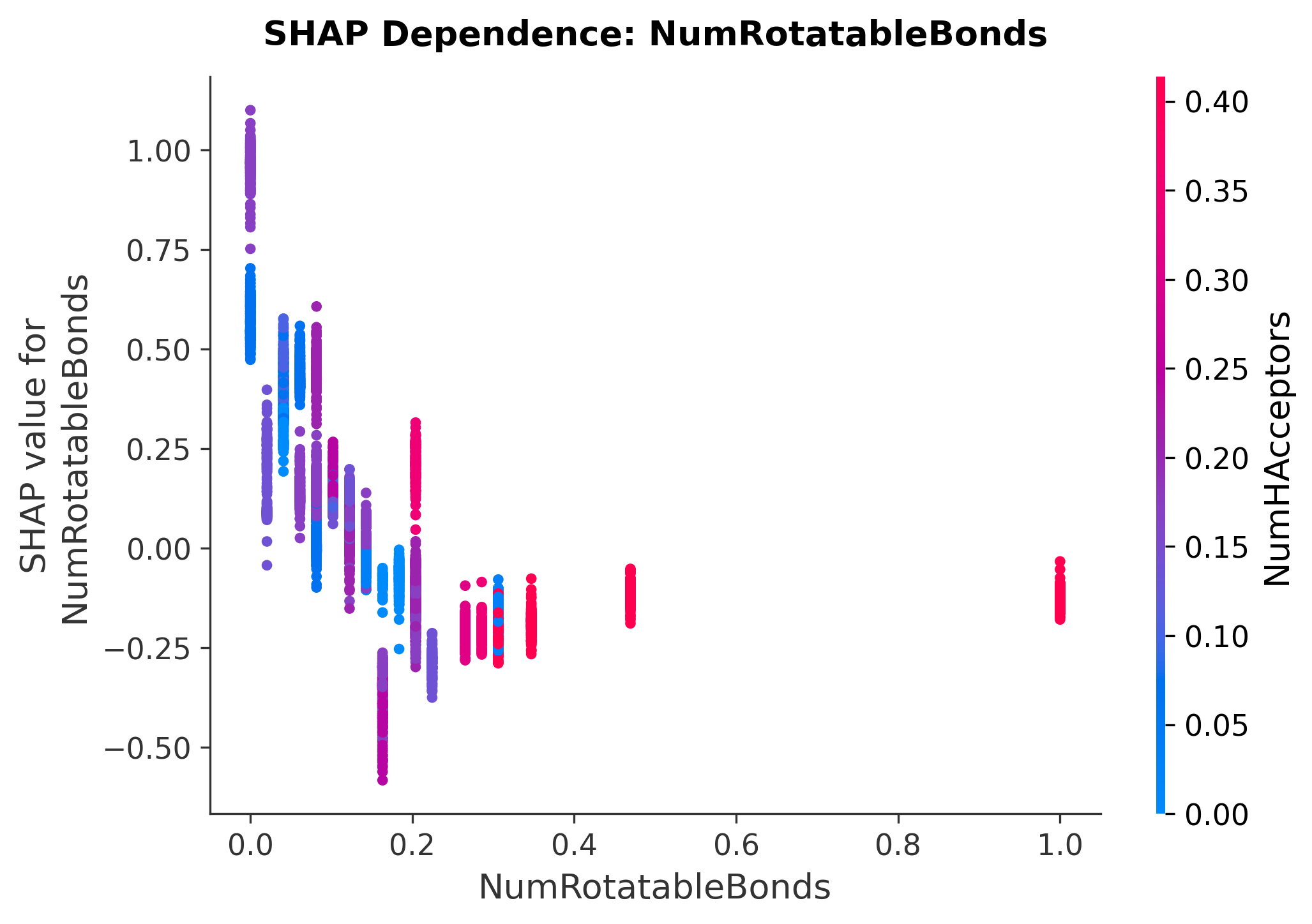

Supplement: Supplementary file 1 [file jox-16-00087-s001.zip › Supplementry_Figures/Figure_S13_RotatableBonds.png]

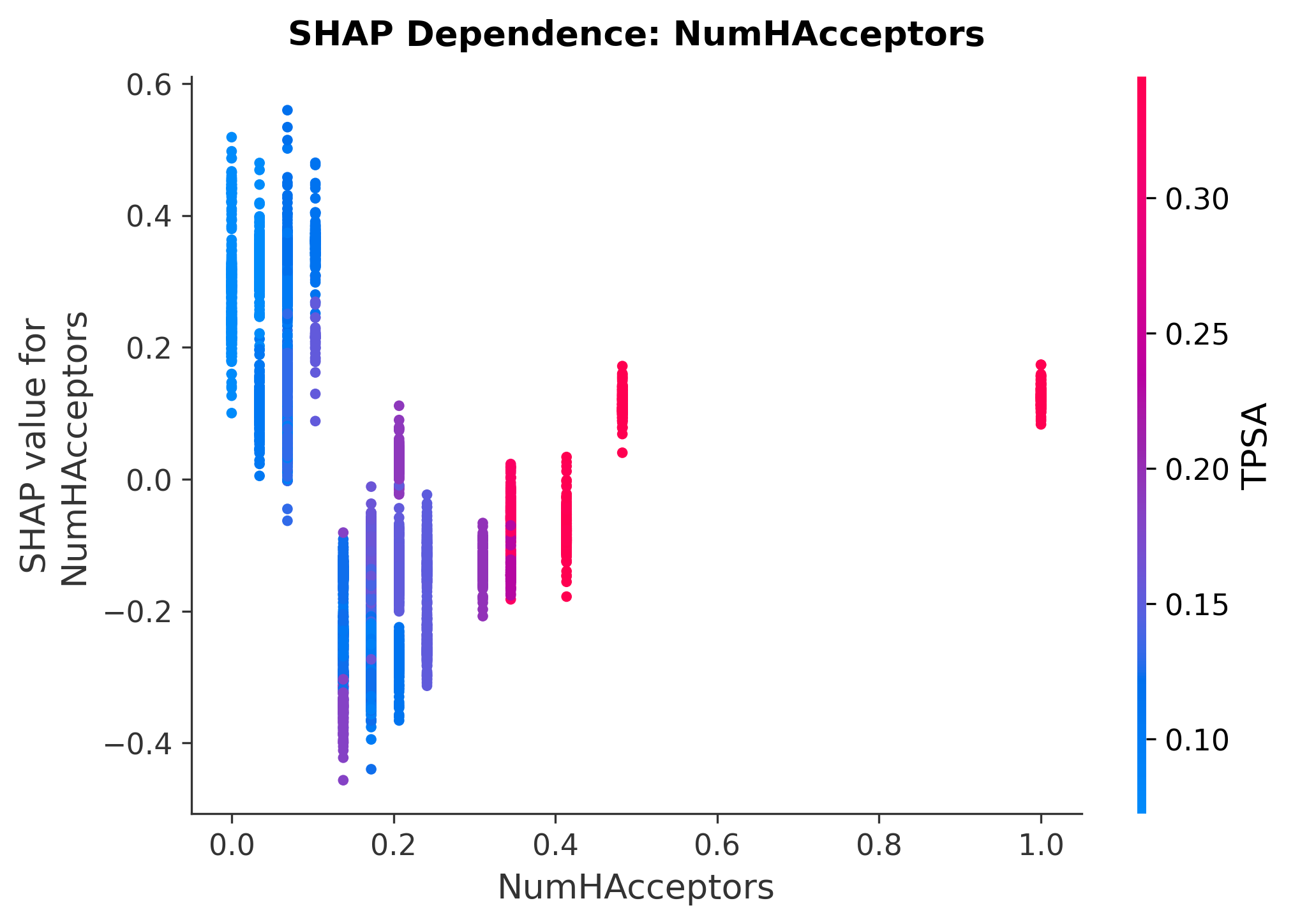

Supplement: Supplementary file 1 [file jox-16-00087-s001.zip › Supplementry_Figures/Figure_S14_HAcceptors.png]

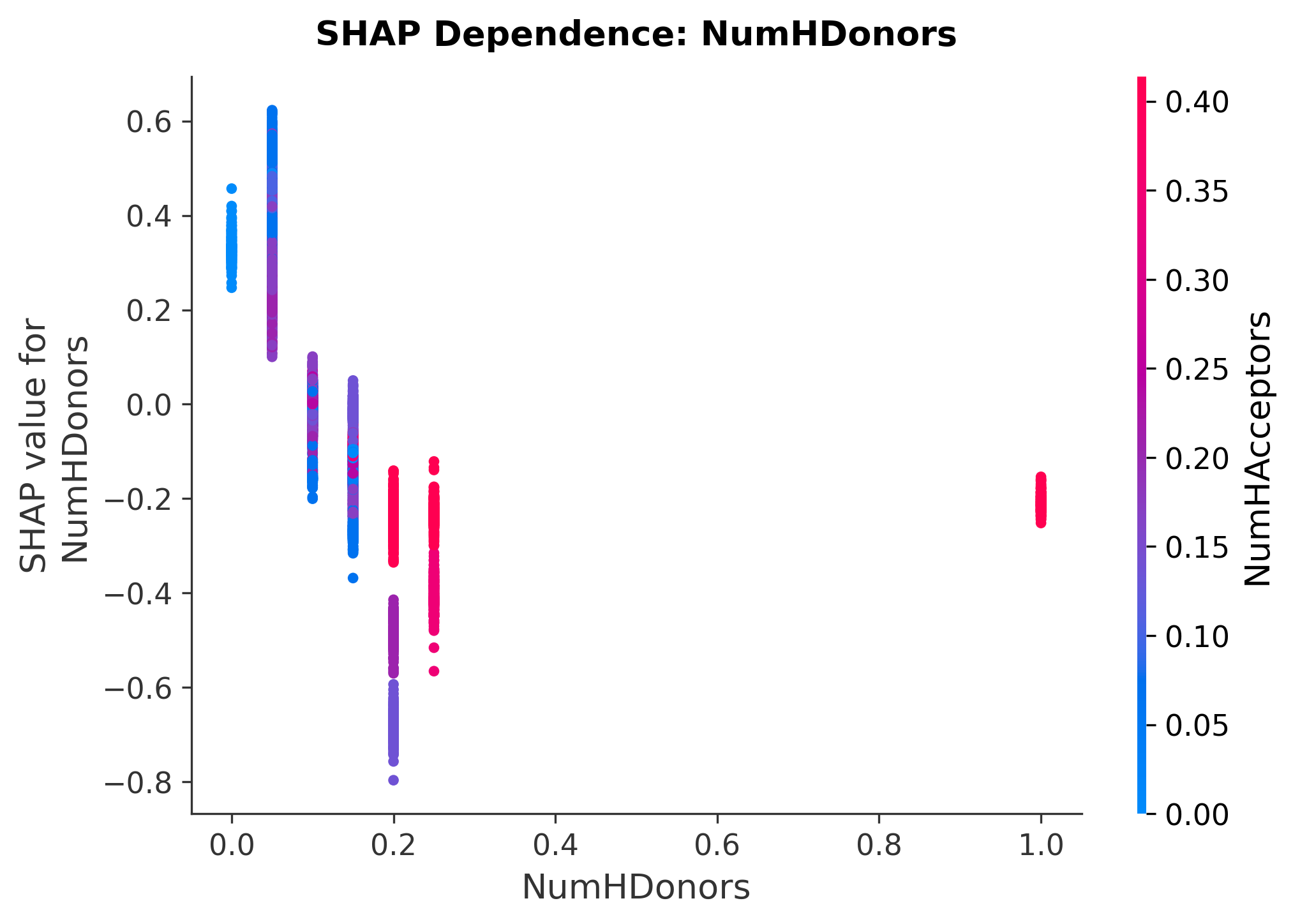

Supplement: Supplementary file 1 [file jox-16-00087-s001.zip › Supplementry_Figures/Figure_S15_HDonors.png]

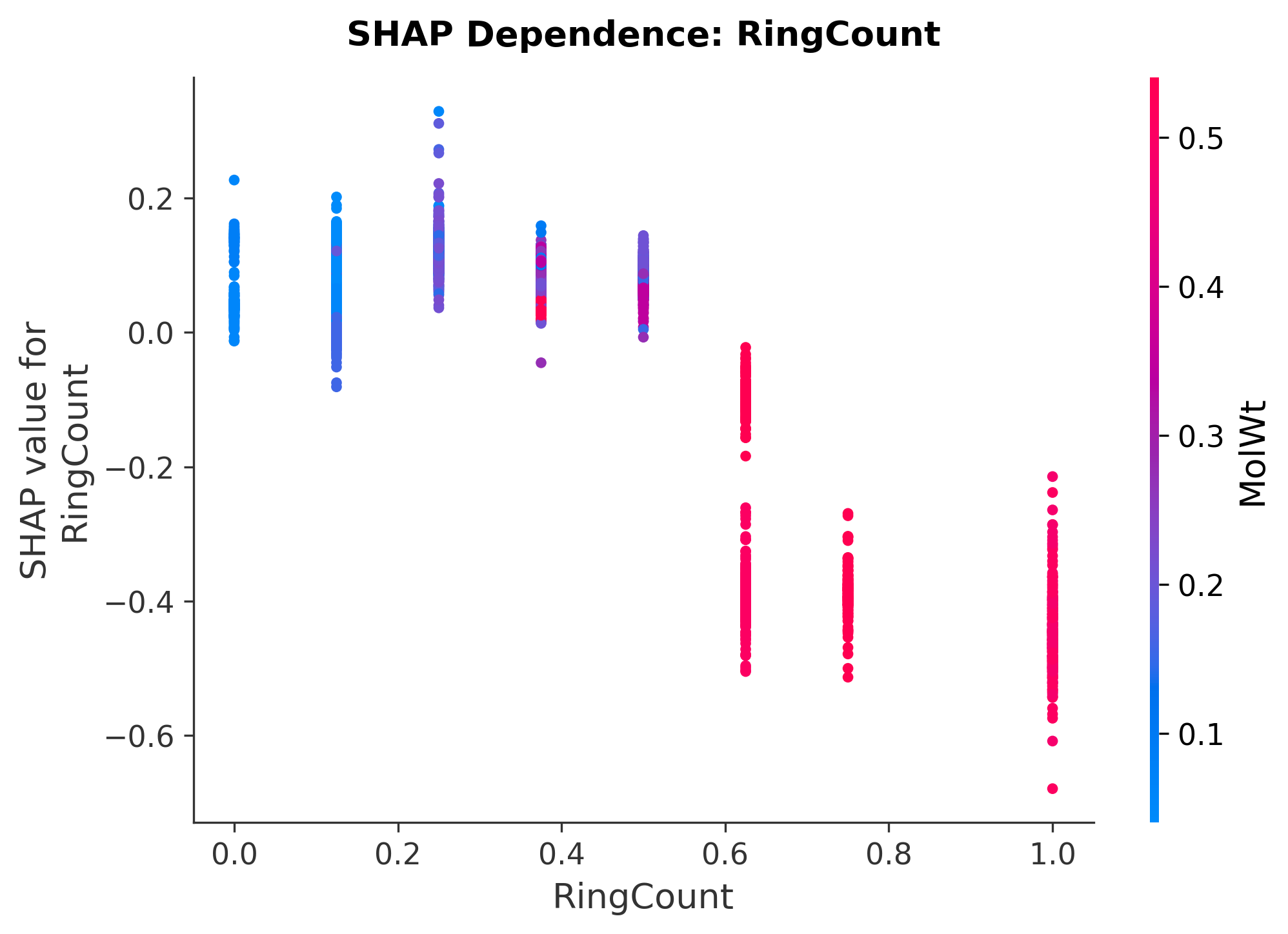

Supplement: Supplementary file 1 [file jox-16-00087-s001.zip › Supplementry_Figures/Figure_S16_RingCount.png]

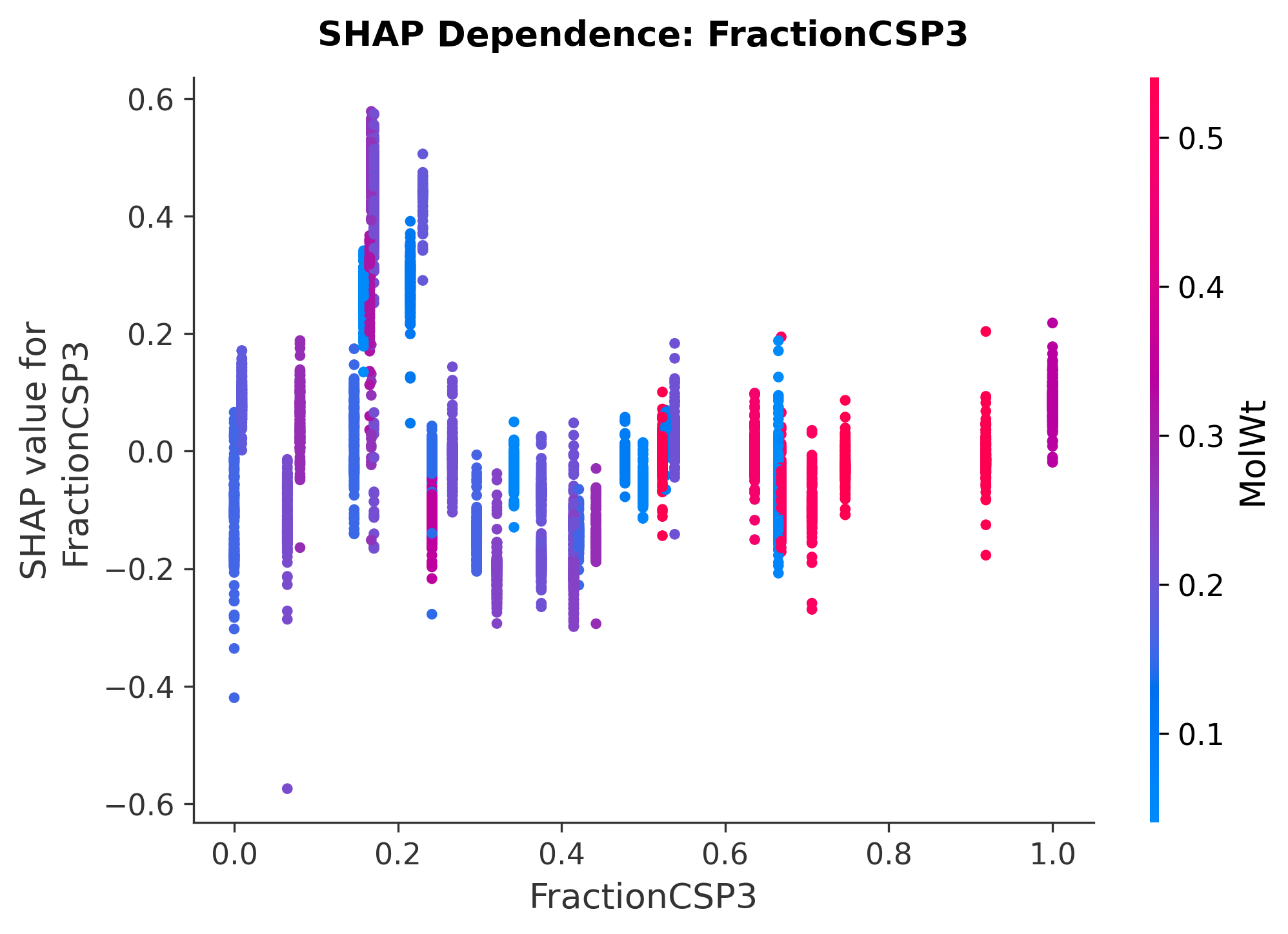

Supplement: Supplementary file 1 [file jox-16-00087-s001.zip › Supplementry_Figures/Figure_S17_FractionCSP3.png]

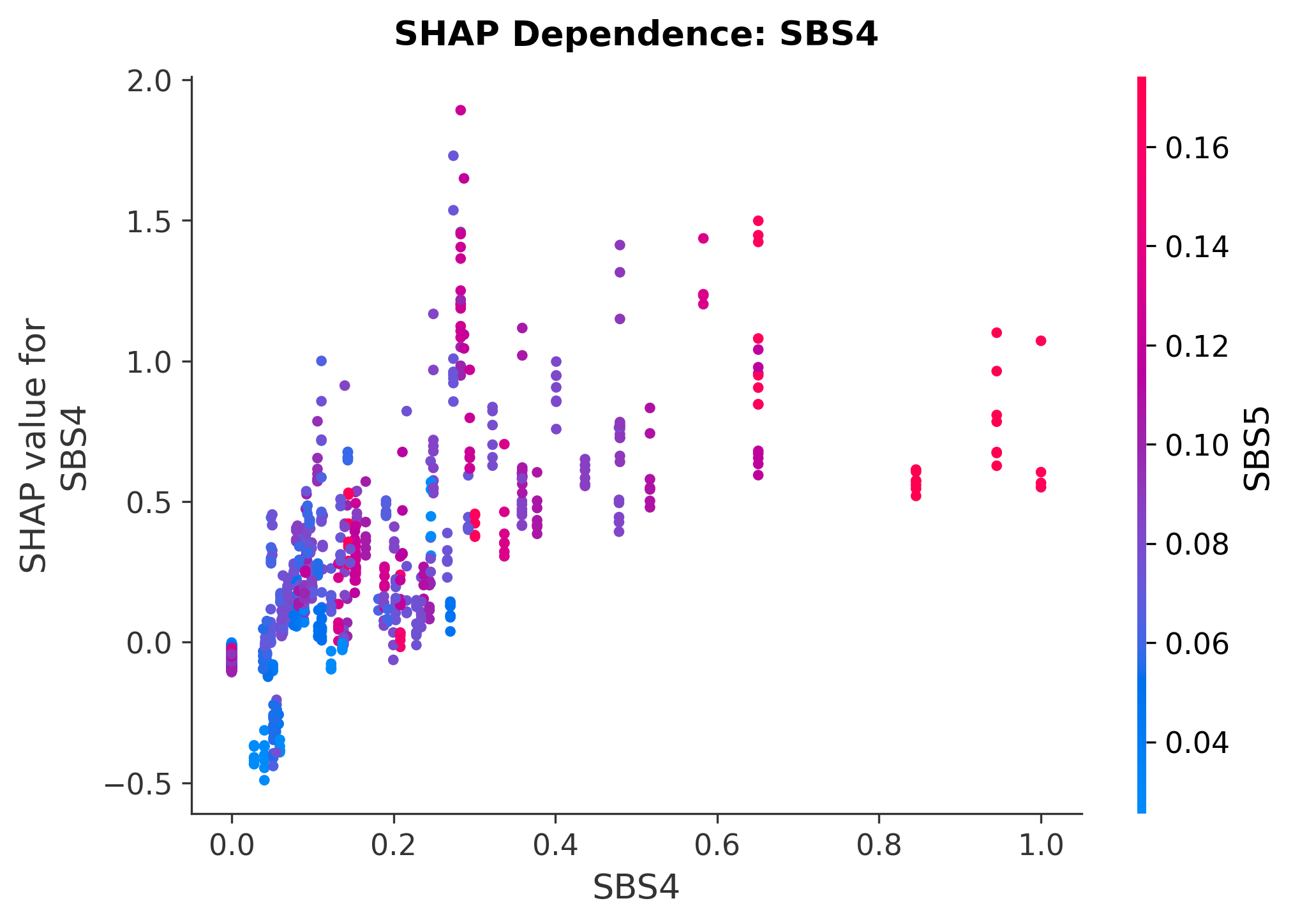

Supplement: Supplementary file 1 [file jox-16-00087-s001.zip › Supplementry_Figures/Figure_S1_SBS4.png]

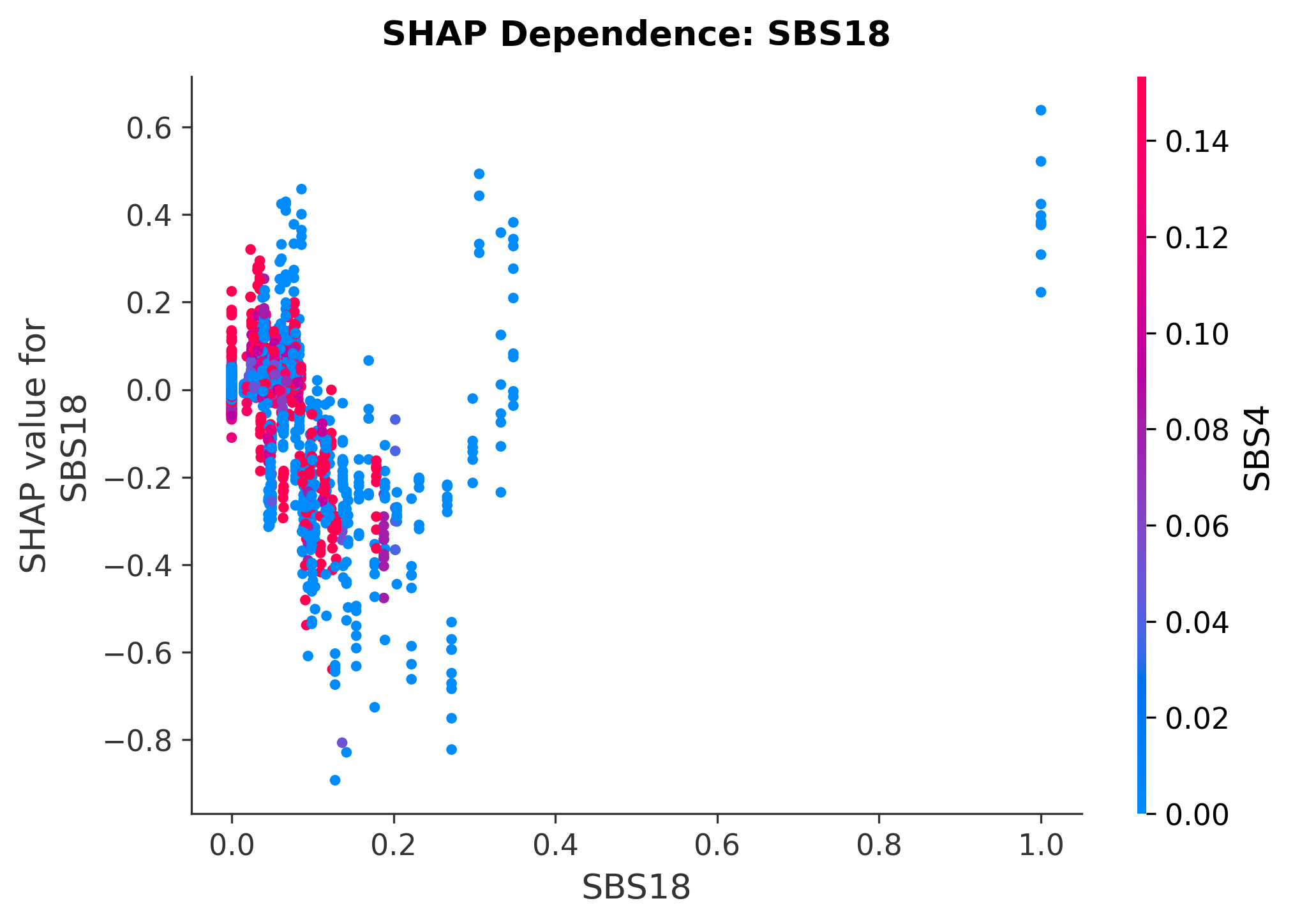

Supplement: Supplementary file 1 [file jox-16-00087-s001.zip › Supplementry_Figures/Figure_S2_SBS18.png]

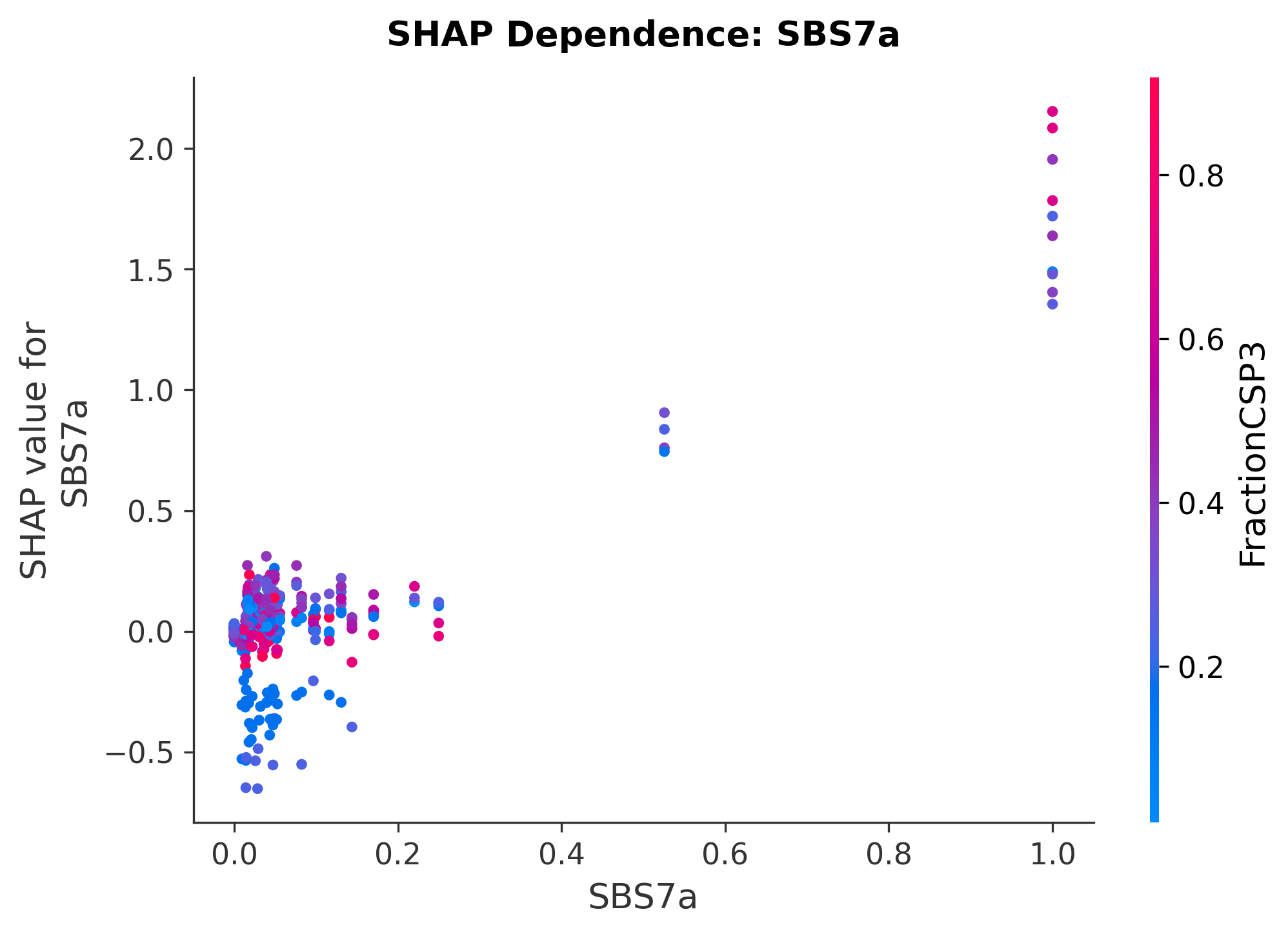

Supplement: Supplementary file 1 [file jox-16-00087-s001.zip › Supplementry_Figures/Figure_S3_SBS7a.png]

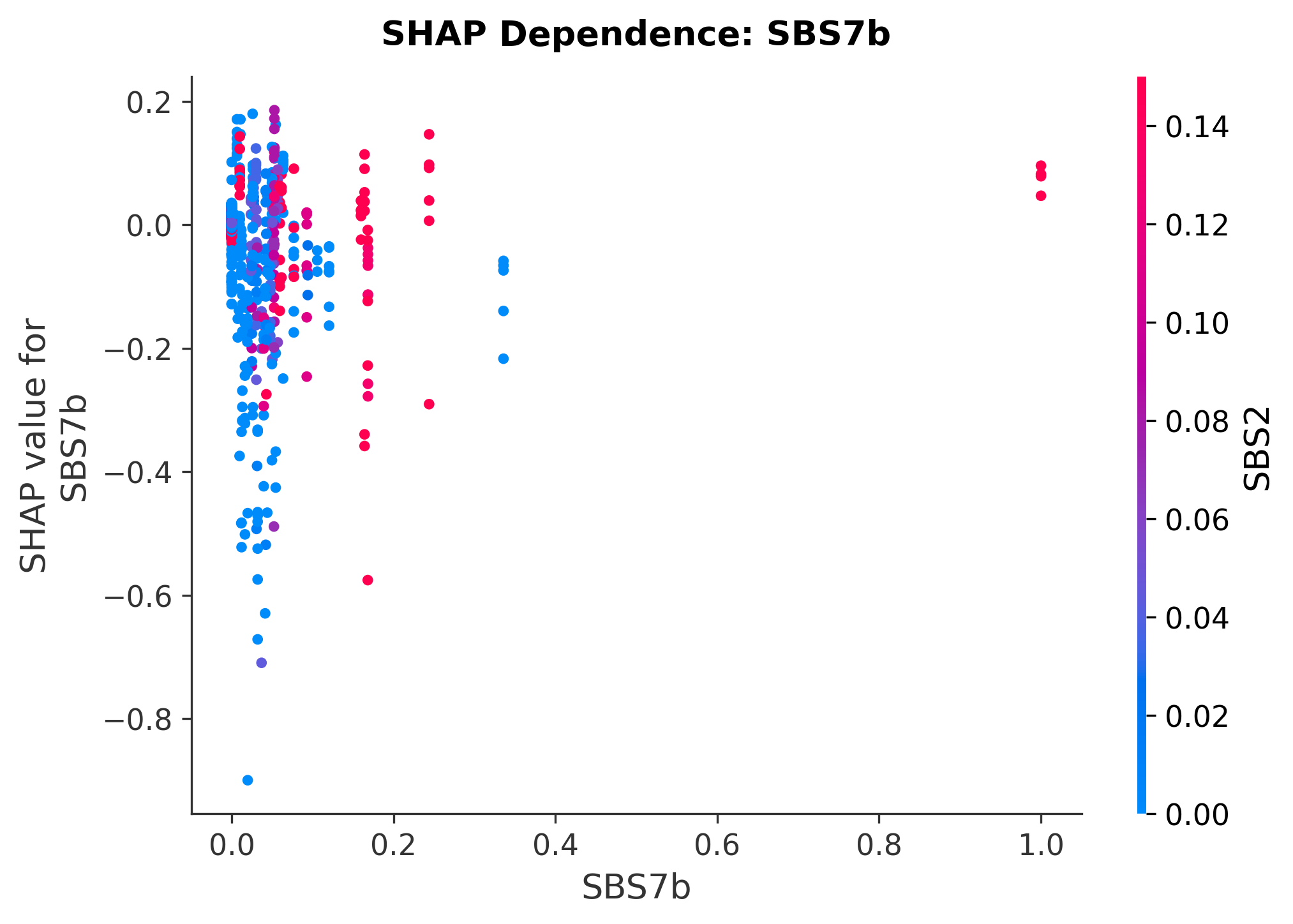

Supplement: Supplementary file 1 [file jox-16-00087-s001.zip › Supplementry_Figures/Figure_S4_SBS7b.png]

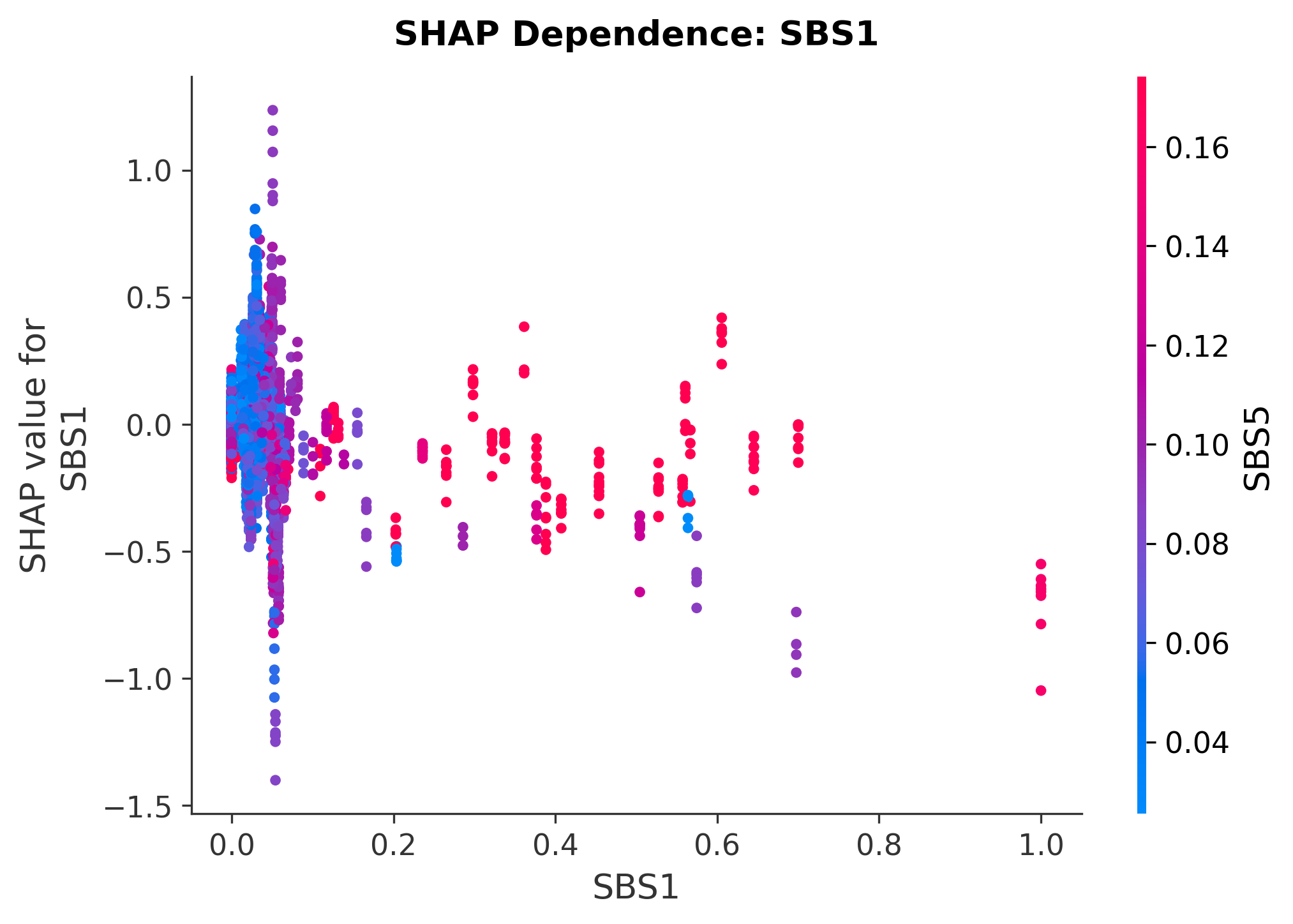

Supplement: Supplementary file 1 [file jox-16-00087-s001.zip › Supplementry_Figures/Figure_S5_SBS1.png]

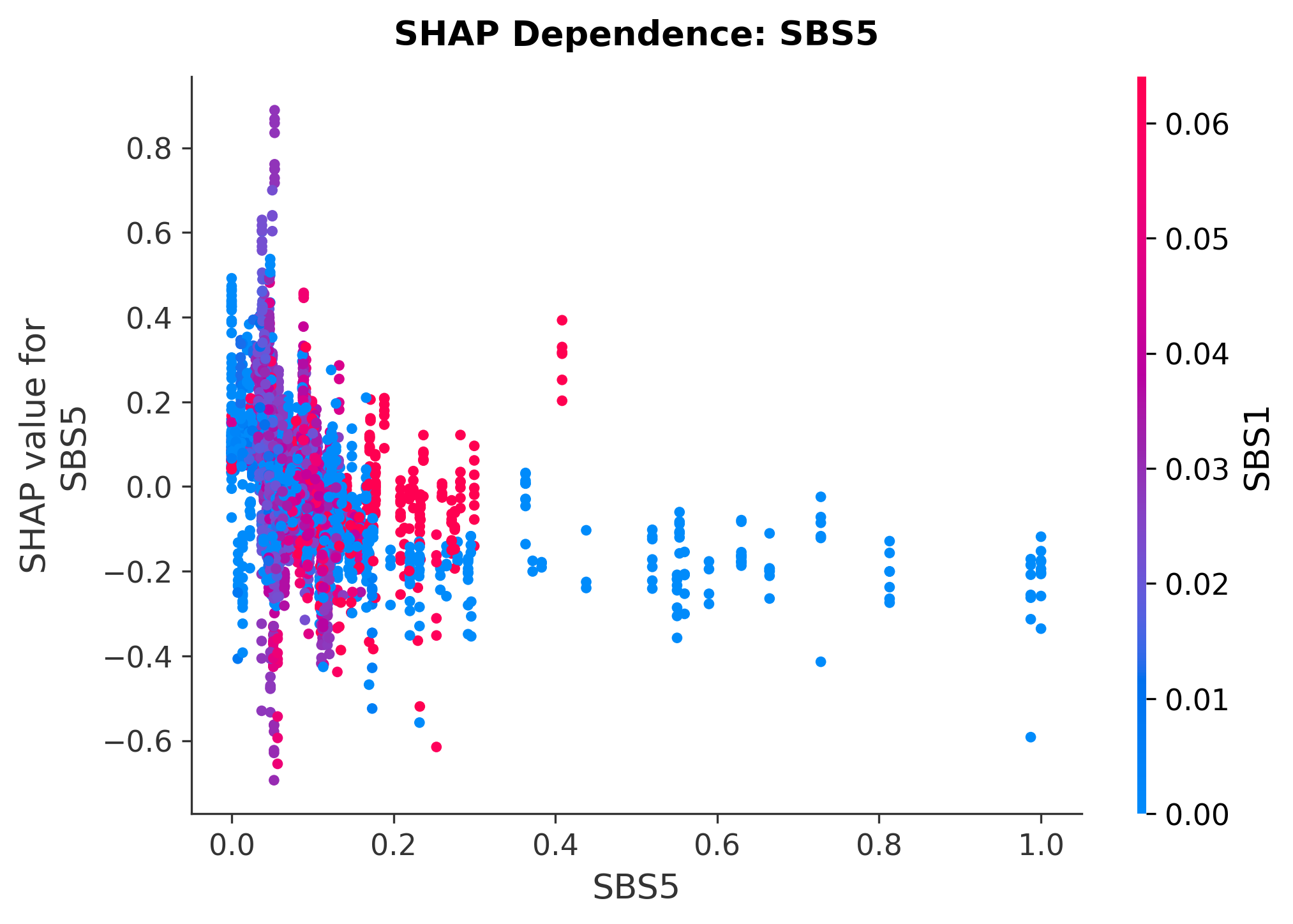

Supplement: Supplementary file 1 [file jox-16-00087-s001.zip › Supplementry_Figures/Figure_S6_SBS5.png]

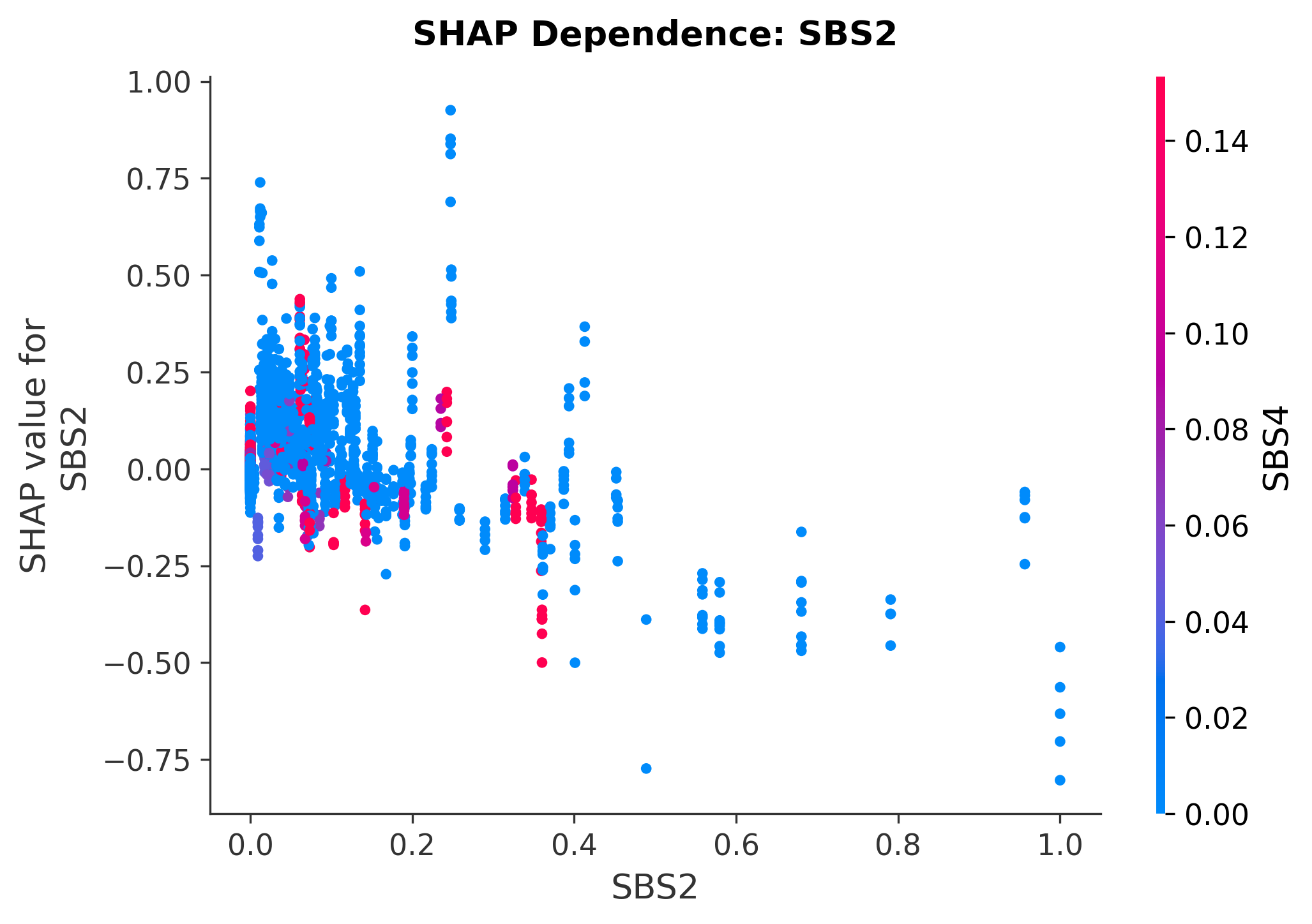

Supplement: Supplementary file 1 [file jox-16-00087-s001.zip › Supplementry_Figures/Figure_S7_SBS2.png]

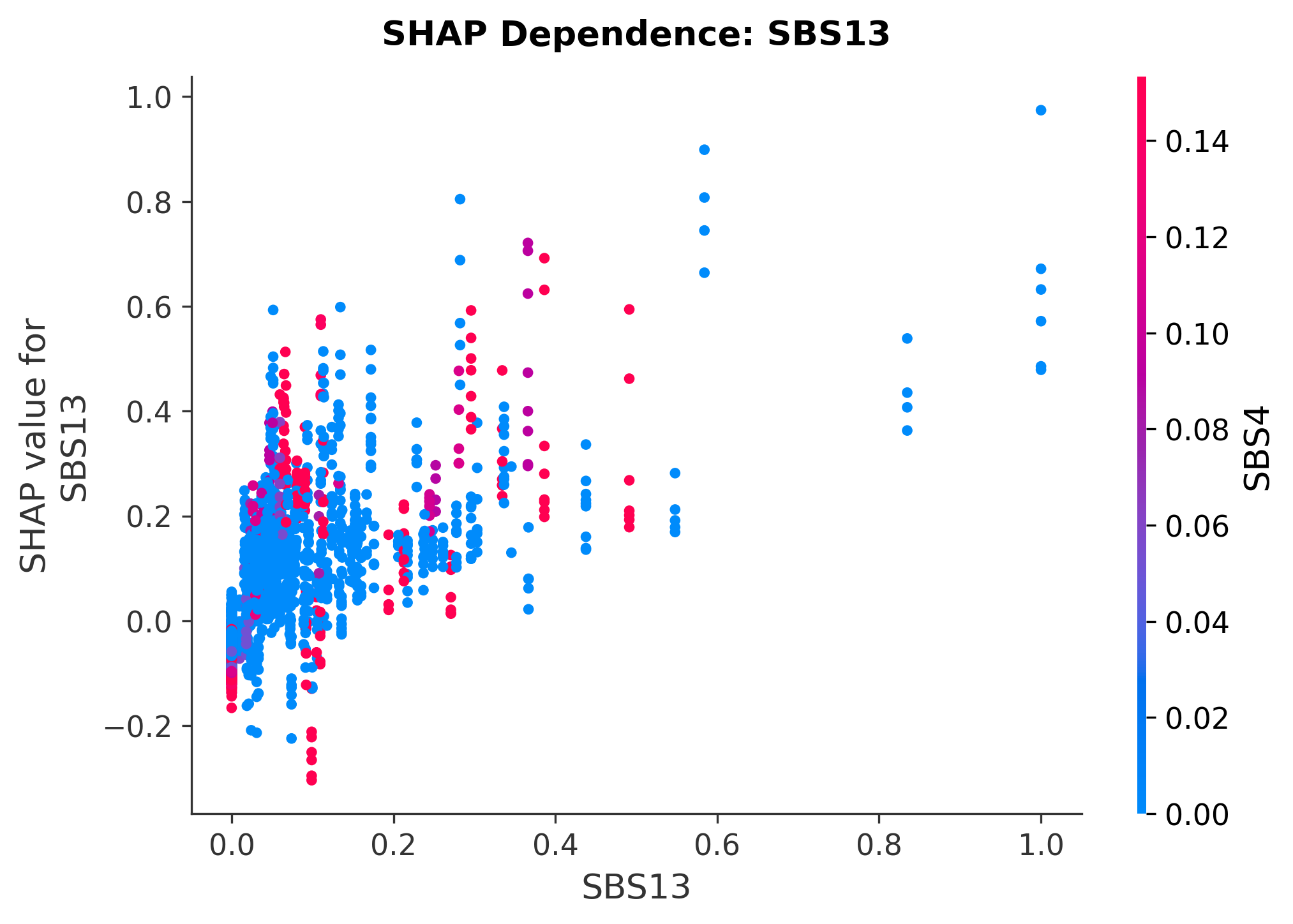

Supplement: Supplementary file 1 [file jox-16-00087-s001.zip › Supplementry_Figures/Figure_S8_SBS13.png]

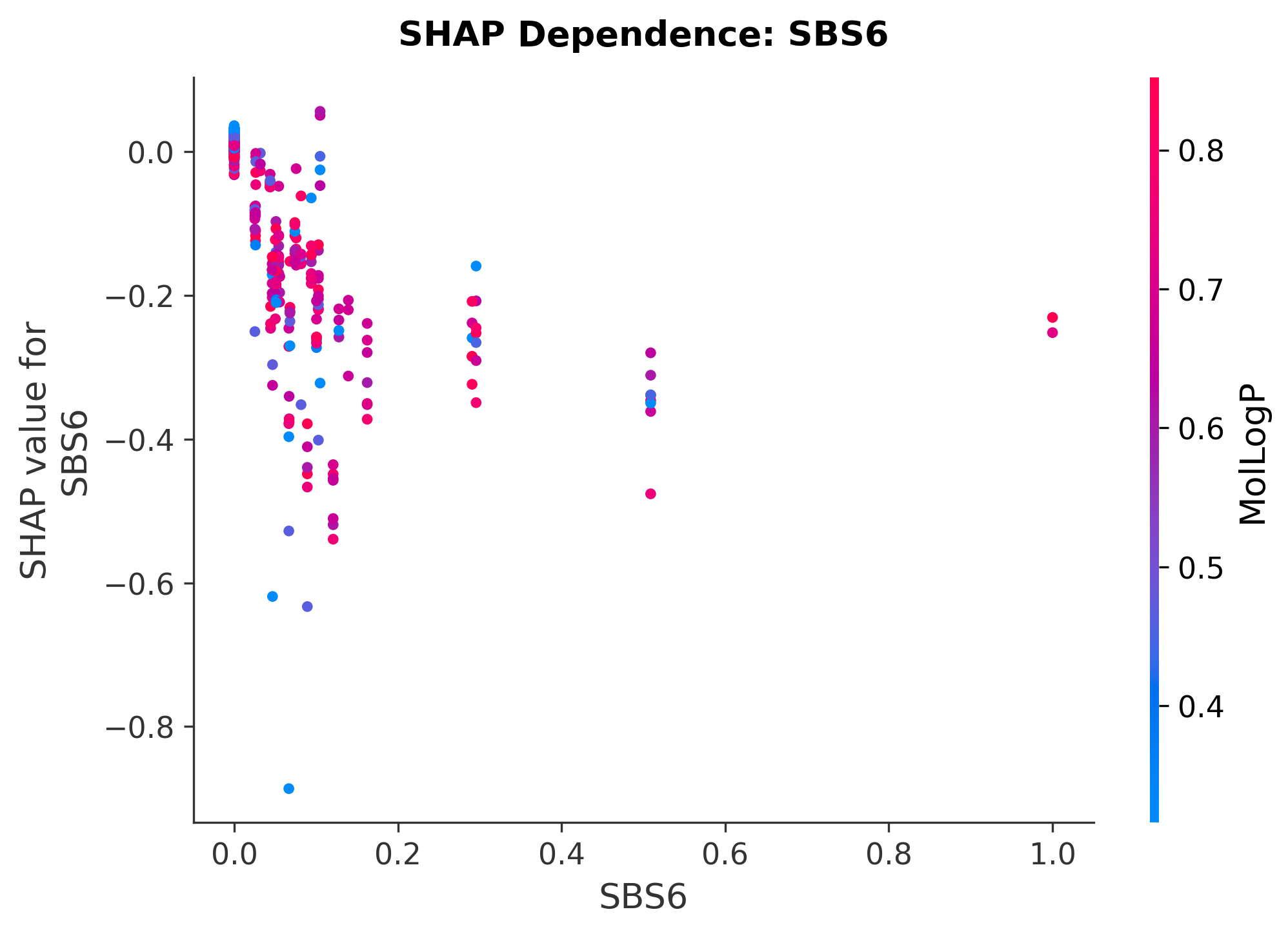

Supplement: Supplementary file 1 [file jox-16-00087-s001.zip › Supplementry_Figures/Figure_S9_SBS6.png]
